# New strategy for drug discovery by large-scale association analysis of molecular networks of different species

**DOI:** 10.1038/srep21872

**Published:** 2016-02-25

**Authors:** Bo Zhang, Yingxue Fu, Chao Huang, Chunli Zheng, Ziyin Wu, Wenjuan Zhang, Xiaoyan Yang, Fukai Gong, Yuerong Li, Xiaoyu Chen, Shuo Gao, Xuetong Chen, Yan Li, Aiping Lu, Yonghua Wang

**Affiliations:** 1 Key Laboratory of Xinjiang Endemic Phytomedicine Resources, Ministry of Education; Pharmacology Department , School of Pharmacy, Shihezi University, Shihezi, Xinjiang, China; 2 Lab of Systems Pharmacology, Center of Bioinformatics, College of Life Science, Northwest A&F University, Yangling, Shaanxi, China; 3 School of Chemical Engineering, Dalian University of Technology, Dalian, Liaoning, China; 4 School of Chinese Medicine, Hong Kong Baptist University, Kowloon Tong, Hong Kong

## Abstract

The development of modern omics technology has not significantly improved the efficiency of drug development. Rather precise and targeted drug discovery remains unsolved. Here a large-scale cross-species molecular network association (CSMNA) approach for targeted drug screening from natural sources is presented. The algorithm integrates molecular network omics data from humans and 267 plants and microbes, establishing the biological relationships between them and extracting evolutionarily convergent chemicals. This technique allows the researcher to assess targeted drugs for specific human diseases based on specific plant or microbe pathways. In a perspective validation, connections between the plant Halliwell-Asada (HA) cycle and the human Nrf2-ARE pathway were verified and the manner by which the HA cycle molecules act on the human Nrf2-ARE pathway as antioxidants was determined. This shows the potential applicability of this approach in drug discovery. The current method integrates disparate evolutionary species into chemico-biologically coherent circuits, suggesting a new cross-species omics analysis strategy for rational drug development.

Despite considerable progress in genome- and proteome-based high-throughput screening methods used for rational drug design, the pharmaceutical industry is not producing new drugs as rapidly as before^1^,^2^. The hope of the rapid translation of ‘genes to drugs’ has foundered on the reality that disease biology is complex, and that drug development must be driven by insights from biological responses^3^. Although the ‘post-genome era’ has resulted in a significant increase in the number of targets of therapeutic interest, most of these targets have no known small-molecule modulators^4^. It is thus urgently needed to select active molecules for specific human diseases from large libraries of chemical molecules.

“Omics” approaches to systems biology have led to the elucidation and analysis of multiple cellular networks representing transcriptional regulation, genetic interactions, protein-protein interactions, and metabolism^5^,^6^. Interestingly, the architecture of these molecular networks from different species are significantly conserved during evolution, an insight that is being used to better define and understand mammalian molecular networks based on homology with their counterparts in lower organisms that were better defined and studied^3^. Therefore, cross-species molecular network homologies may suggest key conserved areas that can be exploited for chemical intervention^3^. For example, previous studies indicated that there are many evolutionarily conserved and functionally convergent molecular subnetworks between humans and model organisms (e.g., mouse, yeast, etc.)^7^,^8^,^9^, some of which are disease-related and can be targeted by many bioactive compounds^10^. In particular, numerous molecules from disparate species such as plants and microorganisms can improve human health and treat various diseases by regulating man’s signaling networks^11^. Thus, it has been hypothesized that different species have evolved conservative modules with similar biological functions and that chemicals may affect them similarly^12^,^13^,^14^,^15^,^16^. For example, a comparative genome analysis revealed that 70% of human cancer genes have orthologues in *Arabidopsis*^17^; and plants and humans can generate structurally similar fatty acid oxidation products in response to wounding (e.g., jasmonic acid in plants and prostaglandins in humans)^15^. However, the underlying mechanisms of these phenomena are unclear^16^. Therefore, determining chemical and biological network associations among different species may assist with understanding mechanism of action of natural products (NPs) and influence the discovery of new chemical entities form an NP library.

Here we present a cross-species molecular network association (CSMNA) profile to define chemical and biological connections between humans and 267 other species of plants, fungi and bacteria; and to identify pharmacologically active chemicals from massive NPs. CSMNA is based on the hypothesis that human and plants/microbes have similar signaling chemicals and biochemical networks in specific functional categories so that molecules produced by different species may overlap functionally (Fig. 1). This technique can be used for targeted screening of bioactive chemicals that regulate certain human pathways via identifying chemico-biologically associated pathways in a specific plant or microbe. CSMNA offers reliable prediction of novel pharmacologically active NPs. With systematic validation, we verified the chemical and biological relationships between the plant Halliwell-Asada (HA) cycle and the human Nrf2-ARE pathway, and how HA cycle related nature chemicals modulate human Nrf2-ARE pathway against oxidative damage.

## Results

### Molecular networks of disparate evolutionary species are structurally and functionally related

Downstream of the transcriptome and proteome, the metabolome is closely related to diversified biological functions^18^,^19^, thus ideal for being applied to establish the chemico-biological association of different species^20^. We manually collected 13,109 functional metabolic network modules for human and 267 other species including 50 plants, 110 fungi, and 107 bacteria^21^ (See § Materials and Methods, Supplementary Table S1). The obtained modules represent tight metabolic units involving 1 to 29 biological reactions (Supplementary Fig. S1).

To search for the chemico-biologically related modules in other species for each human module, we calculated module chemico-biological similarity (MChS) by integrating both metabolic reaction similarities and network topology similarities. This approach can reduce bias caused by genome divergence among different spices, and reveal deep inner biological relationships^22^,^23^. A normalization step is then applied to eliminate module size influence on MChS scoring (§Materials and Methods).

**Figure 2:**
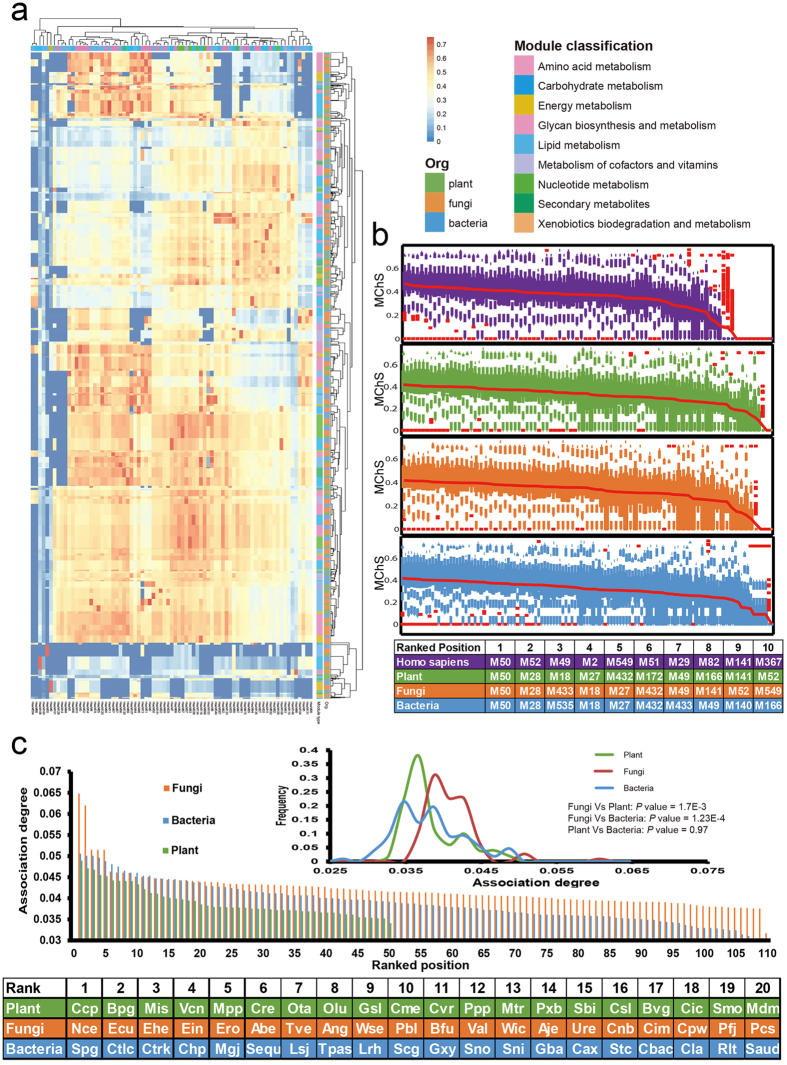
Molecular network of disparate evolutionary species is structurally and functionally related. (**a**) Heat map of cross-species module chemico-biological similarity (MChS) matrix among 86 human modules (columns) and 13,023 plant/microbe modules (rows). Each dot in row i and column j represents MChS score between a plant/microbe module (row i) and a human module (column j). Color code represents MChS scores indicating the degree of chemico-biological similarity of two modules. (**b**) Four MChS score box plots for human, plants, fungi, and bacteria modules. Boxes represent the interquartile range (IQR) between first and third quartiles of MChS scores. Whiskers denote lowest/highest values within 1.5 × IQR from the first and third quartiles, respectively. Red dots represent outliers beyond the whiskers. The red line in each box plot represents MChS score means. The bottom table shows the top 10 human, plants, fungi and bacteria modules with the highest mean MChS scores. (**c**) Chemico-biological association degree of 267 organisms in plant/microbe with human. Association degree is quantified as the ratio of high chemico-biologically similar module pairs (MChS > 0.6) to all module pairs between each organism in plant/microbe and human. The bottom table shows the top 20 plants, fungi and bacteria with the highest chemico-biological association degree with human, respectively. Inset shows ratio distributions. *P*-values indicate statistical significance of the difference of the average ratios (Student’s t-test).

The resulting cross-species MChS matrix represents the association degree of modules between humans and other species (Fig. 2a). Interestingly, modules from different species that carry out the same biological processes tend to cluster together. For example, the “glutathione biosynthesis” modules of 135 plant/microbe species are located in one group (Supplementary Fig. S2). The average MChS scoring for each module (Fig. 2b) shows that M50, guanine ribonucleotide biosynthesis, has the highest module similarity in both humans and plants/microbes. In addition, human M52 (pyrimidine ribonucleotide biosynthesis) and plant M28 (ornithine biosynthesis) also have high module similarity with other modules (Fig. 2b).

Next, we calculated the ratio of highly associated module pairs (MChS > 0.6) to all module pairs for every plant/microbe and human combination. Fungi have the closest association with humans compared with plants and bacteria (Fig. 2c). Average ratios between humans and plants, fungi, and bacteria are 0.0392, 0.0417, and 0.0392, respectively (*P*-value < 0.01, Student’s t-test, Fig. 2c), suggesting that molecular networks are more evolutionarily conserved between fungi and humans. This result is consistent with previous phylogenetic analyses indicating that animals and fungi are closely related and plants constitute an independent evolutionary lineage^24^,^25^. This result also suggests that CSMNA can extract evolutionary relationships among organisms from different kingdoms, supporting our hypothesis that there are conserved molecular modules that provide convergent structures and functions in different species.

### Natural chemical molecules from disparate evolutionary species show similar biological functions

To determine which plant/microbe modules produce or are involved in producing the natural chemicals, we performed attribution analysis of these molecules based on existing biosynthesis information (§Materials and Methods). The most available 2,067 NPs were assigned to the 13,023 plant/microbe modules, each of which contained 8 to 403 NPs (Supplementary Fig. S3). Similarly, all 1,713 drugs were mapped to 76 human metabolic modules (Supplementary Fig. S3) based on the available drug-target interactions^26^,^27^ (§Materials and Methods).

**Figure 3:**
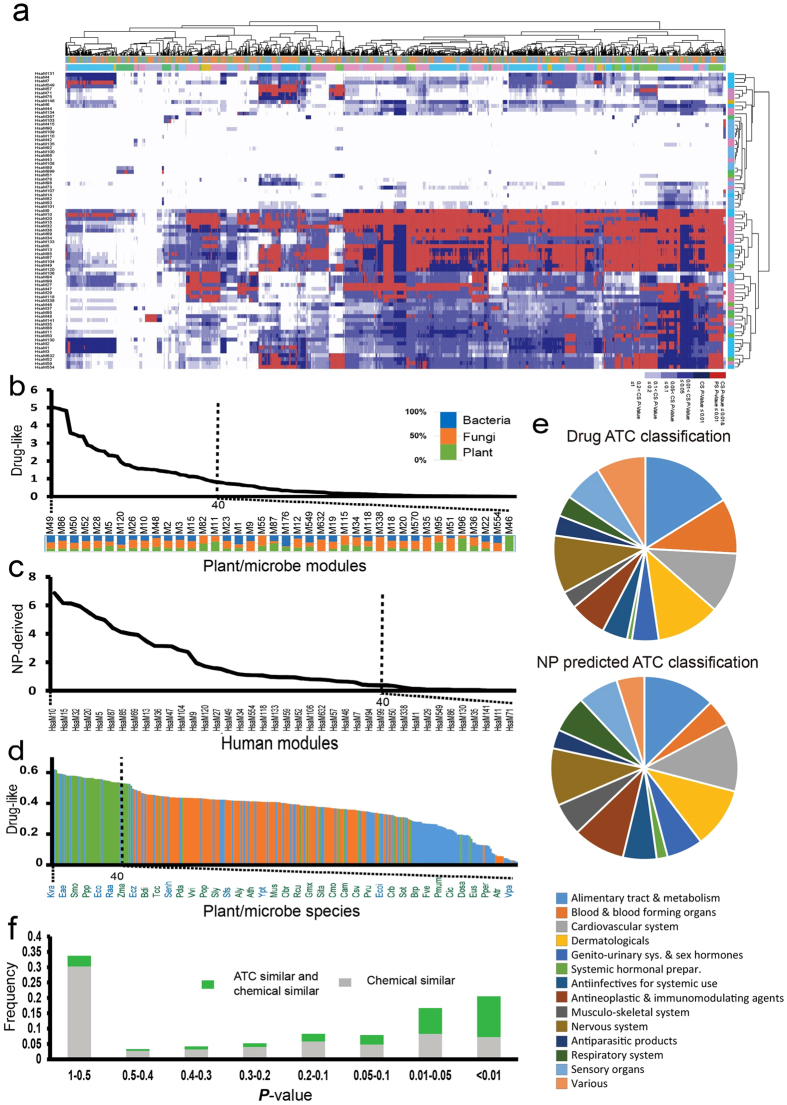
Natural chemical molecules from disparate evolutionary species show similar biological functions. (**a**) Chemical similarity between natural product (NP) sets (columns) and drug sets (rows) from plant/microbe and human modules. Each dot in row i and column j represented chemical structure similarity between a drug set (row i) and a NP set (column j). Color code represents chemical similarity scores indicating the degree of similarity of two compound sets. (**b**) Drug-likeness evaluation for NPs related plant/microbe modules. (**c**) Proportion of NP-derived drugs to all drugs related to each human module. (**d**) Potential of an organism in plant/microbe to produce drug-like NPs. (**e**) ATC classification for drugs and predicted ATC classification for NPs. (**f**) Distribution of chemical similarity score between NP and drug sets, and the proportion of NP and drug sets that have significant ATC similarities (green part).

We first assessed chemical similarity between the NP and drug sets to uncover potential connections between their respective modules. A weighted-ensemble similarity approach was employed to calculate the overall similarity for any two sets of small molecules^28^ (§Materials and Methods). We observed that many chemical molecules corresponding to modules from humans and other species were chemically similar (*P*-value < 0.01) (Fig. 3a, Supplementary Fig. S4). Drug-like NPs tend to distribute in specific modules of a plant or microbe, not randomly (Fig. 3b). For example, 4 of 57 NP sets in *Ricinus communis* are chemically similar (*P*-value < 0.01) with drug sets targeting the ‘glycosaminoglycan biosynthesis (linkage tetrasaccharide)’ module in humans, and these NPs are mainly related to ‘nucleotide metabolism’ modules (Supplementary Fig. S4). In addition, different species have different abilities to produce bioactive NPs. For example, plants, fungi and bacteria have an average of 60, 25, or 9 drug-like NP sets respectively, suggesting that plant NPs are the greatest potential source of new drugs (Fig. 3c,d).

To investigate whether those NP and drug sets with high chemical similarities (*P*-value < 0.01) are also pharmacologically similar, we calculated anatomical therapeutic chemical (ATC) code similarities between the two chemical sets (Fig. 3e). Surprisingly, 65% of the compound sets had significant ATC similarities (*P*-value < 0.01, Fig. 3f). For instance, 27 NPs of SmoM570 (isoleucine biosynthesis) were chemically similar with 35 drugs of HsaM133 (polyamine biosynthesis) (*P*-value = 1.2 × 10^−5^); and they also had significant pharmacological similarity in the treatment of liver damage (*P*-value = 1.6 × 10^−4^)^29^. Importantly, among the top 25 pharmacologically similar NP sets, 29% are clinical drugs as annotated in KEGG Drug, Drugbank or TTD databases (*P*-value = 2.5 × 10^−6^, Fisher’s exact test, Supplementary Table S2).

These data proved a close relationship between NPs and drug molecules from two aspects. Chemical molecules produced by a module of particular plants and microorganisms could be closely related to drug molecules interacting with the specific human module. Chemically, these two sets of molecules have structural similarities and are pharmacologically, functionally consistent. These findings again suggested that natural chemical molecules having functional convergence characteristics across disparate evolutionary species may play similar biological functions.

### New drug discovery based on the chemico-biological association between different species

**Figure 4:**
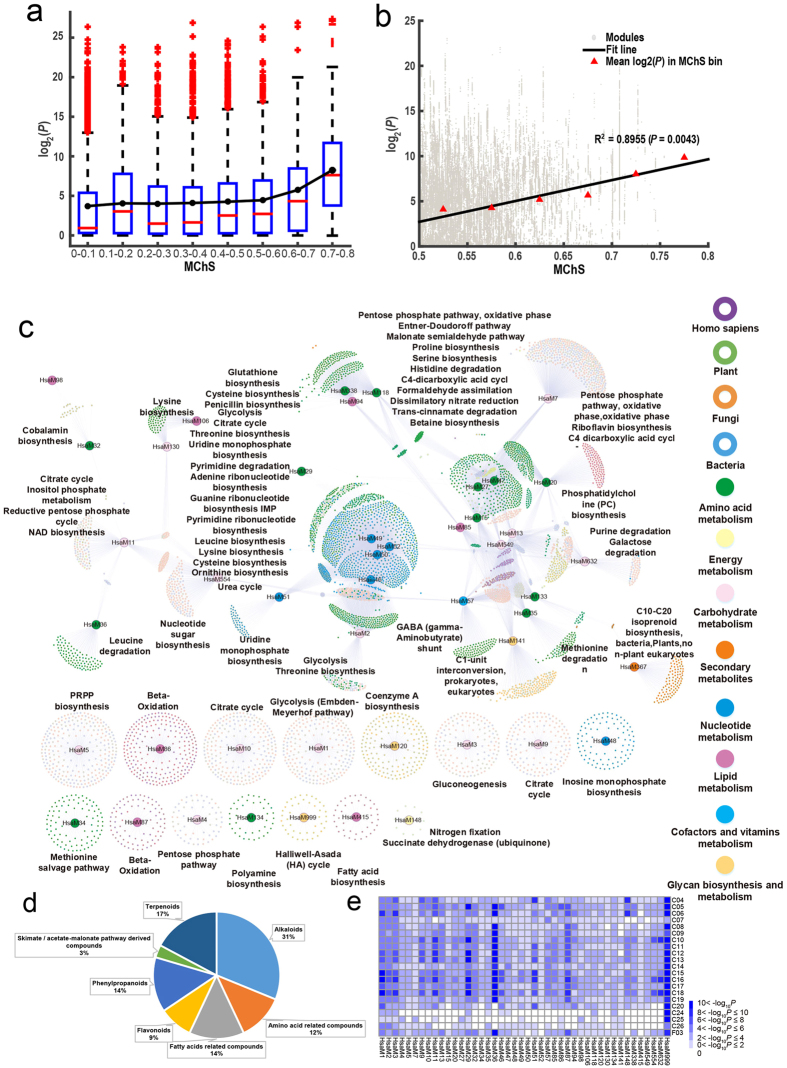
New drug discovery based on the chemico-biological association between different species. (**a**) Box plot for MChS score between human and plant/microbe modules and chemical similarity between NP and drug sets. (**b**) Correlation analysis between MChS score and chemical similarity for module pairs with MChS score >0.5. (**c**) Module-module relation network (MMN) between human and other species. Module pairs with MChS score >0.6 and with significant chemical similarity (*P*-value < 0.01) was screened out to form this network. (**d**) Main categories of NPs from plant/microbe modules involved in MMN. (**e**) Human module-disease association analysis for human modules involved in MMN.

To investigate whether the chemical similarity between NP and drug sets can be attributed to the chemico-biological association between modules of human and plants/microbes, a box plot analysis was first performed on the two score sets (Fig. 4a). As MChS scores increased from 0.1 to 0.6, the chemical similarity of NP and drug sets did not change significantly. However, the chemical similarity dramatically increases 10^4^-fold as the MChS score increases from 0.6 to 0.8. We calculated the ratio of the number of highly related module pairs (MChS ≥ 0.6) that has similar chemicals to the number of all highly related module pairs (MChS ≥ 0.6). A hypergeometric test was applied to obtain the chance of module pairs with both MChS ≥ 0.6 and highly similar chemicals. We found that 37% highly related human-plant/microbe module pairs have similar chemical sets (*P*-value << 0.01, hypergeometric test). Further, we calculated the correlation coefficient between the chemical similarity and MChS score and found that the chemical similarity between NP and drug sets was highly correlated with MChS (R^2^ = 0.9, *P*-value < 0.01, Fig. 4b). Thus, for plant/microbe modules that are highly chemico-biologically associated with certain human module, their associated NPs can interact with the human module.

Based on this, we then searched for NPs that can be used to treat a particular disease. In total, 12,007 module pairs consisting of 43 human modules and 5,000 non-human modules were extracted with MChS score >0.6 and with significant chemical similarity (*P*-value < 0.01) (Fig. 4c). Relationships between modules and diseases were obtained from disease-gene associations (§Materials and Methods). The 2,463 NPs were mapped to 387 human disease entities through 43 human functional modules. These NPs mainly included alkaloids (31%), terpenoids (17%), and flavonoids (9%) (Fig. 4d). As shown in Fig. 4e, human modules were associated with 21 disease types. For instance, neoplasms (C04) were associated with 6 different molecular modules/networks. Interestingly, modules of carbohydrate metabolism were connected to the maximum categories of NPs, and were involved in the most disease types. For example, the 233 NPs produced by *Thiobacillus denitrificans’* module “glycolysis” were enriched in N07X (nervous system drugs). Associated human modules have been confirmed to involve neurological symptoms as well^30^.

These data suggest that chemical molecules corresponding to chemically and biologically conserved molecular networks between different species are highly related. Investigation into the relationships of molecular networks between humans and other species and natural molecules corresponding to molecular networks of other species will assist us with drug discovery for compounds in great demand.

### Natural products related to the plant HA cycle can modulate the human Nrf2-ARE module

**Figure 5:**
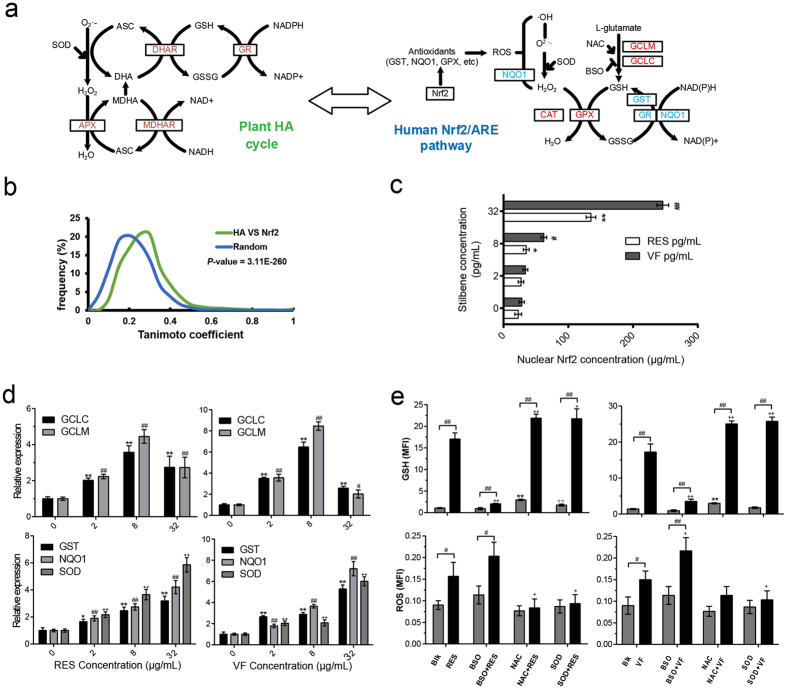
Plant HA cycle related nature chemicals modulate the human Nrf2-ARE module. (**a**) Illustration of the chemico-biological similarity between plant HA cycle and the human Nrf2-ARE module. (**b**) Structure similarity between chemicals related to plant HA cycle and human Nrf2-ARE module. (**c**) Nuclear Nrf2 increased with resveratrol and α-viniferin. (**d**) Quantitative RT-PCR confirmation of induction of key downstream genes of the Nrf2-ARE pathway (GCLC, GCLM, GST, SOD and NQO1). (**e**) Cellular glutathione (GSH) and reactive oxygen species (ROS) increased with resveratrol and α-viniferin compared with controls. *, ^#^, ^+^indicate *p* < 0.05; **, ^##^, ^++^indicate *p* <0.01 (two-tailed Student’s t-test; n ≥ 3). Errors bars are standard deviations of measurements.

To verify the reliability of this method, we selected a representative human Nrf2-ARE module and its associated module: HA cycle of plants, which have a strong chemico-biological association (MChS score = 0.61) and a pharmacological relationship (*P*-value = 4 × 10^−3^) (Fig. 5a). The HA cycle is the main metabolic pathway in plants for resisting oxidative damages caused by various abiotic and biotic stresses via hydrogen peroxide (H_2_O_2_) detoxification^31^. While the human Nrf2 module is needed to protect cells from oxidative stress, and dysfunction of this pathway is noted in cancers, neurodegenerative diseases and cardiovascular diseases^32^.

Surveying the literature, we collected 155 NPs whose synthases are regulated by the HA cycle (Supplementary Table S3). Structural similarity between chemicals related to the plant HA cycle and the human Nrf2-ARE module is significantly greater than random (*P*-value < 0.01, Fig. 5b). To investigate which of these NPs can directly target the human Nrf2-ARE pathway, we predicted multiple ligand-target interactions for the 24 proteins involved in the Nrf2 pathway using systems drug targeting (SysDT)^33^ and weighted ensemble similarity (WES)^28^. This process revealed 16 druggable proteins interacting with 95 HA cycle-related NPs (Supplementary Table S4). Then a cellular thermal shift assay (CETSA) was established to validate the 41 drug-target interactions between 10 hub proteins on the Nrf2 pathway and 41 HA cycle-related NPs (Supplementary Table S5)^34^. We observed that 24% (10/41) of the predicted drug-target interactions were valid (Supplementary Fig. S5).

Next, resveratrol and α-viniferin were studied to identify detailed regulatory functions in an Nrf2-ARE module in human leukemic K562 cells. Gene expression profile analysis indicated that differentially expressed genes (DEGs) were significantly enriched in the pathway “oxidative stress-induced gene expression via Nrf2” (Supplementary Table S6). In particular, antioxidant proteins such as GSTM3, GCLC, NQO1, HSPA1A, TXNIP, AKR1C1, and EPHX1 were also markedly up-regulated, providing necessary cellular protection in response to xenobiotics, heavy metals, and UV light^35^,^36^. Specifically, nuclear Nrf2 increased in a dose-dependent manner in K562 cells after resveratrol or α-viniferin treatment (Fig. 5c). We next measured whether increased nuclear Nrf2 affected steady-state glutamate-cysteine ligase catalytic (GCLC) and its modifier (GCLM) subunit. For the 8 μg/ml resveratrol-treated K562 cells, GCLC and GCLM were statistically significantly higher than controls (20 ± 8% and 100 ± 14%, respectively) (Fig. 5d). Additionally, expression of the other three Nrf2-regulated antioxidant enzymes, i.e., NAD(P)H quinone oxidoreductase 1 (NQO1), glutathione S-transferase family (GST), and superoxide dismutase (SOD), increased in the presence of resveratrol in a dose-dependent way (Fig. 5d). Thus, resveratrol increased nuclear Nrf2 and elevated key downstream gene expression in K562 cells. Similarly, α-viniferin also increased expression of downstream genes of Nrf2 (Fig. 5d).

To determine whether resveratrol and α-viniferin can modulate the Nrf2-ARE pathway against oxidative damage in K562 cells, intracellular glutathione (GSH) and reactive oxygen species (ROS) were measured with CMF and DCF fluorescence and both increased with increasing resveratrol in a dose-dependent manner (Fig. 5e). Although pre-treatment with N-acetylcysteine (NAC) or SOD for 12 h increased intracellular GSH by 360% and 250%, respectively, GSH can be increased significantly further by resveratrol by 8 or 9 fold. In contrast, resveratrol reduced damage from BSO (an inhibitor of GCL) treatment of cells as demonstrated by GSH content variations from 90 to 230% between resveratrol-treated and untreated samples (Fig. 5e). Similarly, α-viniferin also alters intracellular GSH and ROS (Fig. 5e). Thus, NPs related to the plant HA cycle can modulate the Nrf2-ARE pathway in human K562 cells.

Taken together, the plant HA cycle and the human Nrf2-ARE pathway may share similar anti-oxidant mechanisms, which results in NPs related to the HA cycle can modulate the human Nrf2-ARE pathway.

## Discussion

Although vast amounts of biological data are available, there is still the matter of how to use them to promote and accelerate drug development. To develop new drugs, one can start from a disease and look for natural products that can be used as drugs to treat it, or can start from a compound and try to identify the disease that it can target by understanding its pharmacological activity. However, because of limited availability of disease models, the technical difficulties of compound extraction, and other related technical problems, investigators are largely working in a blind fashion during the drug development process. Numerous natural products derived from plants and microbes have been successfully developed into drugs, but it is not known why products from such remotely related species can both modulate human physiological networks and benefit human health.

Herein, we established the chemico-biological associations among molecular networks of humans and 267 plants/microbes for targeted drug screening from natural sources. Approximately 1,400 functional module pairs were identified as being significantly associated and these associated pathway pairs have significant biochemical and functional similarities, chiefly involved in carbohydrate, lipid, nucleotide, and amino acid metabolism in different species. Of the pathways, in humans, the most were attributed to carbohydrate metabolism (50%), nucleotide metabolism (25%), and amino-acid metabolism (20%), which are implicated in cardiovascular diseases^37^, cancer^38^, and obesity^39^. Likewise, many plants have myriad of primary/secondary metabolites related to these metabolic processes and these NPs mediate plant defenses against pathogens and stress through signaling and inducing production of “pathogenesis-related” proteins^40^.

By mapping NPs and drugs into functional modules, we further found correlations between pharmacological relevance of NP and drugs and functional associations between humans and other species. This analysis suggests that many NPs (based on CSMNA profiles) may be promising candidates for treating a variety of diseases. These data also confirm that CSMNA, as a systems-based approach, can dissect complex regulatory circuits that govern secondary metabolism responsible for producing specific bioactive substances.

In addition to providing information about pathways associated with the synthesis of NPs, CSMNA can be used to explore drug mechanisms of action and target proteins of associated human disease networks, offering a direct cross-species associated molecular network to identify compounds with the most novel target selectivity. The greatest benefit of this method is the targeting of specific NPs and search for molecular pathways to infer possible treatments for the human body as we elucidate NP activity and pharmacological functions.

Specially, we analyzed both the human Nrf2-ARE pathway and the plant HA cycle to test whether HA-related NPs could target the human Nrf2-ARE pathway. Biologically, the network topology architecture of the plant HA cycle is similar to the human Nrf2-ARE module; enzymes involved in the human Nrf2-ARE pathway share high homology and GO function similarities with plant HA cycle enzymes. Chemically, nearly two-thirds of stress-induced compounds (including both substrates and products) in the plant HA cycle also overlapped or had high structural and functional similarities with known human Nrf2-ARE pathway activators/inhibitors. In addition, and most importantly, 76% of HA-related NPs could target the enzymes involved in the human Nrf2-ARE pathway. Finally, evidence of target validation indicates that plant NPs such as sulforaphane, curcumin, epigallocatechin-3-gallate, resveratrol, cafestol, and kahweol can also target the human Nrf2-ARE pathway and may treat diseases. For example, highly expressed in the skin, tongue, and nose^41^, human Nrf2-ARE target short transient receptor potential channel 3 is also a target for plant-derived aromatic agents for skin sensitization such as eugenol^42^, which is produced by the plants HA cycle under UV stress.

The limitation of CSMNA in discovering new drugs is that it cannot capture all scenarios of drug actions, but rather chemicals with conserved biological functions between human and other species. It is true that many natural-derived drugs exert the similar function in both human and their original plants/microbes. For example, vincristine, an alkaloid produced by *Catharanthus roseus* plants in response to stress conditions such as salinity and UV-B light, have proven effective in the treatment of leukemia and lymphoma^43^,^44^. Nevertheless, there are a lot of other drugs whose mechanism actions do not follow this scenario. Therefore, the scenario-specific applicability of CSMNA is to discovering potential effective NPs from plants/microbes whose molecular networks have associations with humans’.

To sum it up, the CSMNA offers us a new way to discover effective NPs from certain living organisms. However, presently, the method is still limited due to data insufficiency. With more data available in the future, CSMNA will provide a new chance for synthetic biologist to manipulate and re-engineer the molecular network in plants/microbes for yielding novel NPs and pharmaceuticals for human health benefit. In addition, given the traditional Chinese medicine (TCM) as a great resource of bioactive NPs, CSMNA will also help us elucidate the essence of TCM biological effects from an evolutionary and ecological perspective and facilitate the modernization of TCM.

## Methods

### Mining and compiling functional modules

Data for 666 biological modules from humans, plants, fungi, and bacteria were downloaded from the KEGG MODULE database^21^. This biological module represents a tight functional unit of molecules that generally correspond to a specific function in the KEGG pathway map. There are four types of KEGG modules: pathway modules, structural complexes, functional sets, and signature modules. We only used data for pathway modules because the others did not include metabolic reactions. In addition, we collected from the literature pathway modules that are absent from the database. The final dataset comprises 13,109 metabolic modules from human and 267 other species. Each module is described by a combination of enzyme ortholog entries (identified by K number in the KEGG Orthology (KO) database) and a set of biochemical reaction entries (identified by R number in the KEGG REACTION database). For enzymes without species annotations in the KEGG database, we added species information from other databases or from the literature.

### Chemico-biological association between modules of human and plants/microbes

To find chemico-biological associations between modules of human and plants/microbes, we calculated the homological similarity between two metabolic pathway modules via the SUBMAP model^23^. Given two metabolic pathways *P* and 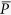 and an upper bound *k* on the size of the connected subnetworks, their homological similarity was calculated when the consistent mapping of the subnetworks of *P* and 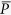 has the maximum similarity. This process was then transformed to an eigenvalue problem. The solution to this eigenvalue problem produced a good integration of metabolic reaction and topological similarities of the subnetworks.

For the metabolic reaction similarity of subnetworks, we first construct three sets for both reaction sets (*R*_*i*_ and 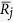). The reaction sets consist of the input compounds (*I*_i_), the output compounds (*O*_i_), and the enzymes (*E*_i_) of the reactions in each subnetwork *R*_i_. Then the similarity of reaction sets *R*_*i*_ and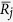 were defined as


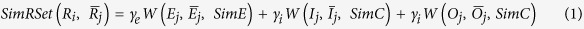


The *W(A, B, SimX)* represent the similarity between two sets A and B with respect to the similarity score *SimX* (*SimE* or *SimC*), where *W* is calculated as the sum of the similarities of the pairs returned by their maximum weight bipartite matching. The *SimE* and *SimC* are two well-known measures reflecting information content similarity for enzyme pairs and SIMCOMP for compound pairs.

The topological similarities of the subnetworks were calculated by following the intuition of IsoRank that if the subnetwork *R*_i_ is mapped to *R*_*j*_, their neighbors in the corresponding pathways should also be similar. The topological similarity was then used to favor mappings of subnetworks that induce similar topologies. The neighborhood definition of reactions were first expanded to reaction subnetworks. Then, the notion of support was generalized to include subnetwork mappings.

Using these similarity values, a vertex weighted graph that connects conflicting mappings with an edge was constructed. Then, the alignment problem is transformed into finding the maximum weight independent set (MWIS) of this graph. A heuristic method was employed to solve the MWIS problem. The result of this method provided us an alignment that has no conflicting pair of mappings (i.e., consistent). Finally, to eliminate the effects of module size to the association score, we normalized association scores by dividing them by the geometric mean of scores obtained from aligning each module against itself.

### Drugs acting on human modules

Chemical, pharmaceutical, and biological information of drugs from DrugBank^26^, KEGG^21^, PubChem^45^ and therapeutic target database (TTD) was retrieved^27^. In total, 1,713 drugs and related targets were collected; and drugs were then mapped to human biological modules if the drug targeted the enzymes in the module.

### Associating NPs with plant/microbe modules

To determine plant/microbe module-related NPs, we first constructed a link between plant/microbe modules and NP biosynthesis pathways based on pathway annotation in KEGG MODULE databases. We then assigned NPs in the biosynthesis pathway to the modules.

### Chemical structure similarity between NP and drug sets

To determine whether NPs assigned to a specific module in plants and microbes were structurally similar to drugs targeting human modules, the weighted ensemble similarity approach was used to calculate the overall similarity between any two compound sets^28^. This technique judges similarities between two sets of ligands even though they share no identical ligand. The parallel approach was demonstrated to be highly accurate in drug targeting and repositioning^28^,^46^.

The similarity between NP sets and drug sets was first approximated by summing the pairwise molecule similarity scores across the ligand sets using CDK fingerprints. Given two sets of compounds, *C* = (*c*_*1*_*,c*_*2*_*,…c*_*n*_) and *C’* = (*c′*_*1*_*,c′*_*2*_*,…,c′*_*m*_), we firstly define a raw similarity score between them *rs*(*C,C′*), which is first approximated by summing the pairwise molecule similarity scores across the ligand sets.


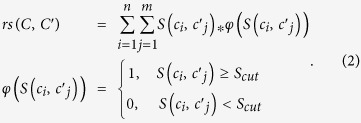


Where the 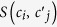 is the Tanimoto similarity of *c*ompound *c*_*i*_ and *c′*_*j*_*. S*_*cut*_ represents the similarity threshold which indicate whether the 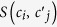 has contribution to the similarity of two compound sets. However, *rs*(*C,C′*) may be subjected to the product of the compound set size (*ps*(*C,C′*) = *n* × *m*). To correct for these drawbacks, we then convert the raw score to a size-bias-free z-score using the mean and standard deviation of raw scores modeled from sets of random molecules.


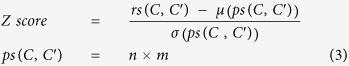


Where *μ(ps(C,C′))* and 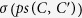 represent the expected mean and standard deviation of 50 raw scores of random compound set pairs with the *ps*(*C*,*C′*) of product size.

The detail processes of *Z score* are as follows:

1. For one product size, construct 50 random compound set pairs and calculate the mean and standard deviation values of raw scores at different similarity thresholds (*S*_*cut*_) where 0 ≤ *S*_*cut*_ < 1 by step size 0.01;
2. Sample at least 5,000 of product size from the range 1 (1 × 1) to 9000(300 × 300), where 1 and 300 are the minimum and maximum set sizes; and repeat the step (1) 5000 times. Therefore, for each *S*_*cut*_, we can obtain 5000 of *μ* and *σ* at different product sizes;
3. For each *S*_*cut*_, plot all *μ* and σ vs. the set size of protein ligand and then the *y*_*μ*_ = *α*_*1*_ + *β*_*1*_ and *y*_*σ*_ = *α*^*r*^_*2*_ + *β*_*2*_ were applied to determine the equations of μ and σ, respectively; Using the fitted *y*_*μ*_ and *y*_*σ*_, transform all random raw scores to the random z scores by formula; Construct a histogram of these z scores and fit the histogram to extreme value type I distributions (EVD). Specially, the threshold fits to an extreme value distribution, which forms the basis of a blast algorithm and sequence similarity
4. Choose the *S*_*cut*_, such that the histogram best fits an EVD based on goodness of fit. *S*_*cut*_ equals 0.51 here.

Finally, we expressed the chemical similarity score between two sets as the probability of a given z-score being higher than that obtained from random data (*P*-value).

### Anatomical therapeutic chemical (ATC) code prediction for NPs

The Anatomical Therapeutic Chemical (ATC) Classification System established by the WHO is used for the classification of drugs. This pharmaceutical coding system categorizes drugs according to the organ or system on which they act and their therapeutic, pharmacological and chemical characteristics. To predict the anatomical therapeutic chemical (ATC) code for NPs, we applied a similarity-based predictor of ATC code database known as SPACE^47^, which was designed to predict drug-ATC class (ATC code) associations. SPACE uses a logistic regression framework to integrate multiple heterogeneous data sources, including chemical structures, target proteins, side-effects, drug-induced gene expression, and chemical-chemical associations, to construct the prediction model. It is suitable for ATC code prediction of new compounds with structural information only. For each compound, SPACE offers predicted candidate ATC codes with a score measuring the possibility of compound-ATC code associations. Compounds with ATC codes whose probability scores >0.8 were selected.

### Pharmacological similarity between NP and drug sets

We calculated the ATC similarity between the NP and drug sets to evaluate their pharmacological similarity. Considering the hierarchical structure of ATC codes, we calculated the similarity scores between NPs and drugs using the semantic similarity algorithm^48^. Specifically, the similarity between two ATC-codes was calculated as follows:


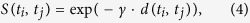


where *d (t*_*i*_*, t*_*j*_) characterizes the shortest distance between ATC codes *t*_*i*_ and *t*_*j*_ in the hierarchical structure of the ATC classification system. *γ* is a pre-defined parameter (set to be 0.25 in this study)^48^.

The ATC similarity between two compounds is then calculated as:


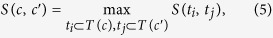


where T(*c*) and T(*c’*) are the ATC sets that compounds *c* and *c*’ belong to, respectively.

ATC similarity between any two compound sets is calculated using the weighted-ensemble similarity approach as described above. The similarity score of ATC codes with the same first class code was set as the threshold.

### Associating human modules with diseases

To characterize module-disease associations, a comprehensive disease-associated gene dataset was built by collecting genes known to be associated with various diseases from DisGeNET^49^ and CTD^50^ databases. Disease and gene information was mapped to MeSH and Entrez Gene ID, respectively, for normalization. Then, normalized disease-gene interactions were integrated and overlapped interactions were manually deleted. Finally, we obtained 28,437 disease-gene interactions including 8,184 genes and 6,337 diseases. We next linked the disease-associated genes to the human module if that gene was a member of that module. Given the two sets of genes in a module and a disease, we counted the number of genes in each of them and the number of their overlapping genes. Then, we calculated the *P*-value for overrepresentation with Fisher’s exact test and corrected the *P*-value by multiple testing. For modules related to diseases assigned to a specific disease category, we selected the minimum *P*-value to reflect the strength of the module-disease category relationship.

### Cell line and cell culture

The human leukemic K562 cell line was obtained from Cancer Cell Repository (Shanghai Cell Bank, Shanghai, China). The K562 cells were cultured in RPMI-1640 (Gibco-BRL; cat no. 31800-022) containing 10% fetal bovine serum (FBS; Israeli; cat no. 1413865) and 100 U/ml each of penicillin and streptomycin. Cells were grown and maintained at 37 °C in a 5% CO_2_ humidified atmosphere.

### Determination of intracellular ROS, GSH

Cells were treated with the indicated chemicals for 2, 4, and 8 h, washed with PBS, and dealt with 20 mM H2DCFDA (Ex/Em = 488 nm/525 nm) or 5 mM CMFDA (Ex/Em = 492 nm/517 nm) at 37 °C for 30 min. After incubation, the cells were washed twice with PBS, then the relative fluorescence intensity was measured using flow cytometry. The ROS and GSH levels were calculated as the mean fluorescence intensity (MFI) per 1,000 non-necrotic cells.

## Additional Information

**How to cite this article**: Zhang, B. *et al*. New strategy for drug discovery by large-scale association analysis of molecular networks of different species. *Sci. Rep*. **6**, 21872; doi: 10.1038/srep21872 (2016).

## Supplementary Material

Supplementary Information

Supplementary Table S1

Supplementary Table S2

Supplementary Table S3

Supplementary Table S4

Supplementary Table S5

Supplementary Table S6

## Figures and Tables

**Figure 1:**
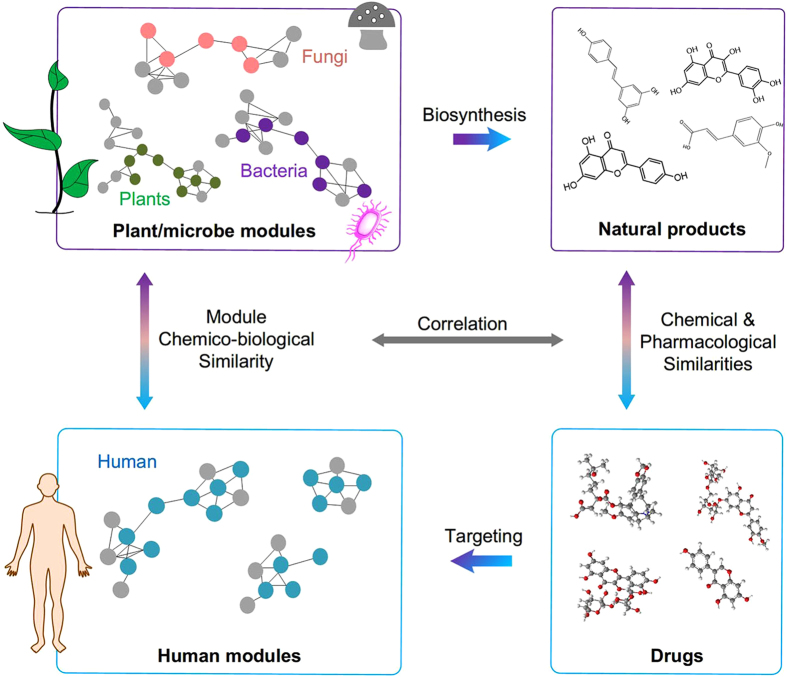
An illustration of the hypothesis that the intrinsic chemico-biological link between human and plants/microbes explains why natural products have pharmacological functions. Natural products (NPs) are produced by specific metabolic pathways of plants/microbes and some of them can be used as drugs to modulate molecular networks of human. We hypothesize the reason of why these NPs can serve as drugs is because their corresponding biosynthesis pathways are chemico-biologically associated with the human molecular networks. Here, a module chemico-biological similarity (MChS) is defined to evaluate the association degree between the human and plant/microbe modules. The similarity between NPs and drugs was assessed with both the chemical structure similarity and pharmacological similarity.

## References

[b1] Csermely P., Korcsmaros T., Kiss H. J., London G. & Nussinov R. Structure and dynamics of molecular networks: a novel paradigm of drug discovery: a comprehensive review. Pharmacology & therapeutics 138, 333–408 (2013). 23384594 10.1016/j.pharmthera.2013.01.016 PMC3647006

[b2] van der Greef J. & McBurney R. N. Innovation - Rescuing drug discovery: *in vivo* systems pathology and systems pharmacology. Nat Rev Drug Discov 4, 961–967 (2005). 16341061 10.1038/nrd1904

[b3] Butcher E. C., Berg E. L. & Kunkel E. J. Systems biology in drug discovery. Nat Biotechnol 22, 1253–1259 (2004). 15470465 10.1038/nbt1017

[b4] Macarron R. . Impact of high-throughput screening in biomedical research. Nat Rev Drug Discov 10, 188–195 (2011). 21358738 10.1038/nrd3368

[b5] Barabasi A. L., Gulbahce N. & Loscalzo J. Network medicine: a network-based approach to human disease. Nat Rev Genet 12, 56–68 (2011). 21164525 10.1038/nrg2918 PMC3140052

[b6] Arrell D. K. & Terzic A. Network Systems Biology for Drug Discovery. Clin Pharmacol Ther 88, 120–125 (2010). 20520604 10.1038/clpt.2010.91

[b7] Sharan R. . Conserved patterns of protein interaction in multiple species. P Natl Acad Sci USA 102, 1974–1979 (2005). 10.1073/pnas.0409522102 PMC548573 15687504

[b8] Berg J. & Lassig M. Cross-species analysis of biological networks by Bayesian alignment. P Natl Acad Sci USA 103, 10967–10972 (2006). 10.1073/pnas.0602294103 PMC1544158 16835301

[b9] Aytes A. . Cross-Species Regulatory Network Analysis Identifies a Synergistic Interaction between FOXM1 and CENPF that Drives Prostate Cancer Malignancy. Cancer Cell 25, 638–651 (2014). 24823640 10.1016/j.ccr.2014.03.017 PMC4051317

[b10] Kapitzky L. . Cross-species chemogenomic profiling reveals evolutionarily conserved drug mode of action. Mol Syst Biol 6 (2010). 10.1038/msb.2010.107 PMC3018166 21179023

[b11] Harvey A. L., Edrada-Ebel R. & Quinn R. J. The re-emergence of natural products for drug discovery in the genomics era. Nat Rev Drug Discov 14, 111–129 (2015). 25614221 10.1038/nrd4510

[b12] Kushiro T., Nambara E. & McCourt P. Hormone evolution: The key to signalling. Nature 422, 122 (2003). 12634761 10.1038/422122a

[b13] Schultz J. C. How plants fight dirty - Biochemical ecology. Nature 416, 267–267 (2002). 11907557 10.1038/416267a

[b14] Schultz J. C. & Appel H. M. Cross-kingdom cross-talk: Hormones shared by plants and their insect herbivores. Ecology 85, 70–77 (2004).

[b15] Schultz J. C. Shared signals and the potential for phylogenetic espionage between plants and animals. Integr Comp Biol 42, 454–462 (2002). 21708739 10.1093/icb/42.3.454

[b16] Howitz K. T. & Sinclair D. A. Xenohormesis: Sensing the chemical cues of other species. Cell 133, 387–391 (2008). 18455976 10.1016/j.cell.2008.04.019 PMC2504011

[b17] Jones A. M. . The impact of Arabidopsis on human health: Diversifying our portfolio. Cell 133, 939–943 (2008). 18555767 10.1016/j.cell.2008.05.040 PMC3124625

[b18] Mo M. L. & Palsson B. O. Understanding human metabolic physiology: a genome-to-systems approach. Trends Biotechnol 27, 37–44 (2009). 19010556 10.1016/j.tibtech.2008.09.007

[b19] Robert-Seilaniantz A., Grant M. & Jones J. D. G. Hormone Crosstalk in Plant Disease and Defense: More Than Just JASMONATE-SALICYLATE Antagonism. Annu Rev Phytopathol 49, 317–343 (2011). 21663438 10.1146/annurev-phyto-073009-114447

[b20] Schweiger R., Baier M. C., Persicke M. & Muller C. High specificity in plant leaf metabolic responses to arbuscular mycorrhiza. Nat Commun 5 (2014). 10.1038/ncomms4886 24848943

[b21] Kanehisa M. . Data, information, knowledge and principle: back to metabolism in KEGG. Nucleic acids research 42, D199–D205 (2014). 24214961 10.1093/nar/gkt1076 PMC3965122

[b22] Tabach Y. . Human disease locus discovery and mapping to molecular pathways through phylogenetic profiling. Mol Syst Biol 9 (2013). 10.1038/msb.2013.50 PMC3817400 24084807

[b23] Ay F., Kellis M. & Kahveci T. SubMAP: Aligning Metabolic Pathways with Subnetwork Mappings. J Comput Biol 18, 219–235 (2011). 21385030 10.1089/cmb.2010.0280 PMC3123932

[b24] Baldauf S. L. & Palmer J. D. Animals And Fungi Are Each Others Closest Relatives - Congruent Evidence From Multiple Proteins. P Natl Acad Sci USA 90, 11558–11562 (1993). 10.1073/pnas.90.24.11558 PMC48023 8265589

[b25] Hedges S. B. The origin and evolution of model organisms. Nat Rev Genet 3, 838–849 (2002). 12415314 10.1038/nrg929

[b26] Law V. . DrugBank 4.0: shedding new light on drug metabolism. Nucleic acids research 42, D1091–D1097 (2014). 24203711 10.1093/nar/gkt1068 PMC3965102

[b27] Zhu F. . Therapeutic target database update 2012: a resource for facilitating target-oriented drug discovery. Nucleic acids research 40, D1128–D1136 (2011). 21948793 10.1093/nar/gkr797 PMC3245130

[b28] Zheng C. . Large-scale Direct Targeting for Drug Repositioning and Discovery. Sci. Rep. 5, 11970; doi: 10.1038/srep11970 (2015). 26155766 PMC4496667

[b29] Terai S. . Improved liver function in patients with liver cirrhosis after autologous bone marrow cell infusion therapy. Stem cells 24, 2292–2298 (2006). 16778155 10.1634/stemcells.2005-0542

[b30] Roland B. P. . Triosephosphate isomerase I170V alters catalytic site, enhances stability and induces pathology in a Drosophila model of TPI deficiency. Biochimica et Biophysica Acta (BBA)-Molecular Basis of Disease 1852, 61–69 (2015). 25463631 10.1016/j.bbadis.2014.10.010 PMC4268122

[b31] Halliwell B. The wanderings of a free radical. Free Radical Bio Med 46, 531–542 (2009). 19111608 10.1016/j.freeradbiomed.2008.11.008

[b32] Hayes J. D. & Dinkova-Kostova A. T. The Nrf2 regulatory network provides an interface between redox and intermediary metabolism. Trends Biochem Sci 39, 199–218 (2014). 24647116 10.1016/j.tibs.2014.02.002

[b33] Yu H. . A systematic prediction of multiple drug-target interactions from chemical, genomic, and pharmacological data. PloS one 7, e37608 (2012). 22666371 10.1371/journal.pone.0037608 PMC3364341

[b34] Jafari R. . The cellular thermal shift assay for evaluating drug target interactions in cells. Nature protocols 9, 2100–2122 (2014). 25101824 10.1038/nprot.2014.138

[b35] Dhakshinamoorthy S., Long D. J. & Jaiswal A. K. Antioxidant regulation of genes encoding enzymes that detoxify xenobiotics and carcinogens. Curr Top Cell Regul 36, 201–216 (2000). 10842753 10.1016/s0070-2137(01)80009-1

[b36] Bloom D., Dhakshinamoorthy S., Wang W., Celli C. M. & Jaiswal A. K. Role of NF-E2 related factors in oxidative stress. Cell and Molecular Responses to Stress: Protein Adaptation and Signal Transduction 2, 229–238 (2001).

[b37] Shukla S. K., Gupta S., Ojha S. K. & Sharma S. B. Cardiovascular friendly natural products: a promising approach in the management of CVD. Nat Prod Res 24, 873–898 (2010). 20461632 10.1080/14786410903417378

[b38] Mann J. Natural products in cancer chemotherapy: past, present and future. Nat Rev Cancer 2, 143–148 (2002). 12635177 10.1038/nrc723

[b39] Han L., Kimura Y. & Okuda H. Anti-obesity effects of natural products. Studies in Natural Products Chemistry 30, 79–110 (2005).

[b40] Jeandet P. . Deciphering the Role of Phytoalexins in Plant-Microorganism Interactions and Human Health. Molecules 19, 18033–18056 (2014). 25379642 10.3390/molecules191118033 PMC6271817

[b41] Salat K., Moniczewski A. & Librowski T. Transient Receptor Potential Channels - Emerging Novel Drug Targets for the Treatment of Pain. Curr Med Chem 20, 1409–1436 (2013). 23409716 10.2174/09298673113209990107

[b42] Vriens J., Appendino G. & Nilius B. Pharmacology of Vanilloid Transient Receptor Potential Cation Channels. Mol Pharmacol 75, 1262–1279 (2009). 19297520 10.1124/mol.109.055624

[b43] Ramakrishna A. & Ravishankar G. A. Influence of abiotic stress signals on secondary metabolites in plants. Plant signaling & behavior 6, 1720–1731 (2011). 22041989 10.4161/psb.6.11.17613 PMC3329344

[b44] Idrees M., Naeem M., Aftab T. & Khan M. M. A. & Moinuddin Salicylic acid mitigates salinity stress by improving antioxidant defence system and enhances vincristine and vinblastine alkaloids production in periwinkle [Catharanthus roseus (L.) G. Don]. Acta Physiol Plant 33, 987–999 (2011).

[b45] Wang Y. . PubChem: a public information system for analyzing bioactivities of small molecules. Nucleic acids research 37, W623–W633 (2009). 19498078 10.1093/nar/gkp456 PMC2703903

[b46] Keiser M. J. . Relating protein pharmacology by ligand chemistry. Nat Biotechnol 25, 197–206 (2007). 17287757 10.1038/nbt1284

[b47] Liu Z. Y. . Similarity-based prediction for Anatomical Therapeutic Chemical classification of drugs by integrating multiple data sources. Bioinformatics 31, 1788–1795 (2015). 25638810 10.1093/bioinformatics/btv055

[b48] Wang Y. C., Chen S. L., Deng N. Y. & Wang Y. Network predicting drug’s anatomical therapeutic chemical code. Bioinformatics 29, 1317–1324 (2013). 23564845 10.1093/bioinformatics/btt158

[b49] Bauer-Mehren A., Rautschka M., Sanz F. & Furlong L. I. DisGeNET: a Cytoscape plugin to visualize, integrate, search and analyze gene–disease networks. Bioinformatics 26, 2924–2926 (2010). 20861032 10.1093/bioinformatics/btq538

[b50] Mattingly C., Rosenstein M., Colby G., Forrest J. Jr. & Boyer J. The Comparative Toxicogenomics Database (CTD): a resource for comparative toxicological studies. Journal of experimental zoology. Part A, Comparative experimental biology 305, 689–692 (2006). 10.1002/jez.a.307 PMC1586110 16902965

