# Supplementary material for: New strategy for drug discovery by large-scale association analysis of molecular networks of different species
Source: Sci Rep. 2016 Feb 25;6:21872. doi: 10.1038/srep21872 (PMC4766474; doi:10.1038/srep21872)
Supplement: Supplementary Information [file srep21872-s1.doc]

**Supplementary Materials**

**New strategy for drug discovery by large-scale association analysis of molecular networks of different species**

Bo Zhang^1§^, Yingxue Fu^2§^, Chao Huang^2§^, Chunli Zheng^2§^, Ziyin Wu^2§^, Wenjuan Zhang^2^, Xiaoyan Yang^1^, Fukai Gong^1^, Yuerong Li^1^, Xiaoyu Chen^2^, Shuo Gao^2^, Xuetong Chen^2^, Yan Li^3^, Aiping Lu^4^, Yonghua Wang^2^*

**^1^** Key Laboratory of Xinjiang Endemic Phytomedicine Resources, Ministry of Education; Pharmacology department , School of Pharmacy, Shihezi University, Shihezi, China.

**^2^** Lab of Systems Pharmacology, Center of Bioinformatics, College of Life Science, Northwest A&F University, Yangling, Shaanxi, China.

**^3^** School of Chemical engineering, Dalian University of Technology, Dalian, Liaoning, China.

**^4^** School of Chinese Medicine, Hong Kong Baptist University, Kowloon Tong, Hong Kong.

^§^ Contributing equally to this work.

* To whom correspondence should be addressed. Contact: yh_wang@nwsuaf.edu.cn

**Supplementary Methods**

**Supplementary Figure S1.** Function categories and number distribution of modules from human and plants/microbes.

**Supplementary Figure S2.** Cluster of the module “M118: Glutathione biosynthesis” from 135 different organisms in plant/microbe.

**Supplementary Figure S3.** Drug and NP sets related to modules from human and plants/microbes.

**Supplementary Figure S4.** The chemical structure similarity matrix between drug and NP sets of human and *Ricinus communis* modules.

**Supplementary Figure S5.** The CETSA experiments.

**Supplementary Methods**

**Cellular Thermal Shift Assay (CETSA)**

The ability of compounds to interact with, and thereby stabilize the target in intact cells, was analyzed essentially as described by Molina et al .

**Human Cell Experiment**

**Reagents**

Culture medium (RPMI 1640), 2,7-dichlorodihydro-fluorescein diacetate (DCFH-DA), 2',7'-dichlorofluorescin diacetate (H2DCFDA), 5-chloromethylfluorescein diacetate (CMFDA), dimethyl sulfoxide (DMSO), 3-(4,5-dimethylthiazol-2-yl)-2,5-diphenyl tetrazolium bromide ( MTT ), Hoechst 33258, Gimsa solution were purchased from Sigma Chemical Co. ( St. Louis, MO, USA). Fetal bovine serum (FBS) was purchased from Tianjin Hao Yang Biological Manufacture CO., L td. (Tianjin, China). First Strand cdna Synthesis Kit and cDNA amplification Kit were purchased from Formentas CO., Ltd., (Vilnius, Lithuania). Penicillin and streptomyc were obtained from Shandong Sunrise Pharmaceutical Co., Ltd. (Zibo, China). All other chemicals are of analytical grade and commercially available.

**Cell viability assay**

Cell viability was measured by the 3-(4,5-dimethylthiazol-2-yl)-2,5-diphenyl- tetrazolium bromide (MTT) assay (T Mosmann., 1983). Cells were briefly trypsinized, seeded into 96-well plates at 4 × 10^3^ cells/well, and pre-incubated for 24 h before treatment. The cells were then exposed to different concentrations of α-viniferin, at 37°C. The medium was removed after incubation, and fresh medium containing 10 mL of 5 mg/mL MTT was added. This medium was removed after 4 h and replaced with blue formazan crystal dissolved in 100 mL DMSO. The absorbance at 570 nm was measured using a microplate reader (Thermo Varioskan Flash 3001, USA). The cell inhibition rate was calculated as 100% × (control group A values - experimental group A values) / control group A values.

**Morphological assay**

The morphological changes in the nuclear chromatin of cells undergoing apoptosis were detected by Hoechst 33258 and Gimsa staining. Briefly, K562 cells treated with or without RES and α-Viniferin were collected, washed with PBS. Then the cells were stained using either 10 mg/mL Hoechst 33258 staining or Gimsa staining in PBS. The apoptotic and necrotic cells were recorded by fluorescence microscopy (Axio Observer A1; Zeiss, Germany).

**ELISA assay**

To investigate the effect of RES and α-Viniferin on Nrf2 expression, cells were pretreated with different concentrations of RES (2, 8, 32 µM), and then the cell media were collected. Nrf2 protein concentration was measured using an ELISA (R&D Systems, Minneapolis, MN, USA) according to the manufacturer's instruction.

**Evaluation of Nrf2/ARE and ER stress pathway-related gene expression**

To analyze the expression levels of the ER stress–related genes GCLM, GCLC, GST, NQO 1, SOD (after 24 h of α-viniferin treatment) and Nrf2 downstream target genes (after 4 h of α-viniferin treatment), the cells were treated as indicated, washed with PBS, and collected. RNA was extracted from the cells using EZ-10 Spin Column Total RNA Minipreps Super Kit (Bio Basic, Inc., Markham, CA, USA) according to the manufacturer’s instructions. The RNA quality was evaluated using the A260/A280 ratio and 1.5% agarose gel electrophoresis. Two micrograms of RNA per sample was transformed into cDNA using Moloney Murine Leukemia Virus reverse transcriptase with a First Strand cDNA Synthesis Kit according to the manufacturer’s instructions (Fermentas, Vilnius, Lithuania). Quantitative real-time RT-PCR was performed using a single-tube SYBR Green kit (QIAGEN, Valencia, CA, USA), Rotor Gene Q real-time PCR system (Rotor Gene Q, QIAGEN), and specific primer sets (the same primers used in the conventional RT-PCR). Only the experiments with a distinct single peak with a melting temperature different from that of the notemplate control were further analyzed. The relative amount of target mRNA was calculated by the 2^−△△Ct^ method. For mRNA normalization, glyceraldehyde-3-phosphate dehydrogenase (GAPDH) was used as an endogenous reference gene for each sample.

| **Gene** | **Primer** |
| --- | --- |
| GAPDH | (+)5’-GAC ATC AAG AAG GTG GTG AAG C-3’  (-)5’-GTC CAC CAC CCT GTT GCT GTA G |
| GCLC | (+)5’- ATG ATG CCA ACG AGT CTG AC -3’  (-)5’- CGC CTT TGC AGA TGT CTT TC -3’ |
| GCLM | (+)5’-GAC AAA ACA CAG TTG GAA CAG C -3’  (-)5’-CAG TCA AAT CTG GTG GCA TC-3’ |
| NQO1 | (+)5’-AGA GTG GCA TTC TGC ATT TCT G-3’  (-)5’-CTG GAG TGT GCC CAA TGC TA-3’ |
| GST | (+)5’-ATG GGC CAT GAG CTG TTT CT-3’  (-)5’-AGC CCA GGT ACT CAT GAA CA-3’ |

**DNA microarray procedure**

**RNA extraction and DNA microarray analysis**

SurePrint G3 Human Gene Expression 8×60K v2 Microarray was used to identify genes in K562 cells whose expression was altered by treatment with 10 μM Res for the various time periods using a balanced block design. The microarray analyses were supported by 6 biological replicates. The Invitrogen Super-Script II direct cDNA labeling system was utilized to label cDNAs which were prepared from total RNA in the presence of either Cyanine-3 (Cy3) dCTP or Cyanine-5 (Cy5) dCTP (Perkin Elmer, Waltham, MA). The amounts of dye integrated into cDNA were measured on a NanoDrop spectrophotometer (ThermoFisher Scientific, Pittsburgh, PA). Equimolar amounts of Cy3 and Cy5 labeled cDNAs were combined and added to MWG hybridization solution. This solution remained a constant temperature (95°C) for 3 min., was cooled in ice for 3 min and loaded onto the microarray slide. Hybridizations were exercised for 16 h at 42°C on a GeneTAC hybridization station (Genomic Solutions, Ann Arbor, MI). According to a Genomic Solutions protocol, slides were washed on the automated hybridization station. Then we applied a PerkinElmer ScanArray Express Microarray Scanner to scan the slides. Only probes for which at least three out of six replicates passed the low intensity filter were collected for the analysis.

For the control K562 vs. Res-treated K562 expression profiles, we employed the same balanced block design with a dye swap using six biological replicates. Equimolar amounts of the cDNAs that labeled by Cy3 and Cy5 were added to Agilent Whole Human Genome Arrays, and hybridized for 17 h at 60 °C using a MAUI hybridization system (BioMi-croSystems, Salt Lake City, UT). The microarray slides were then washed using Agilent Gene Expression Wash Buffer and scanned by a Perkin Elmer ScanArray Express scanner.

**Statistical Analysis of Microarray Data**

We used PerkinElmer ScanArray software with the default lowess normalization settings to extract the feature intensities from the scanned image. For control K562 vs. Res-treated K562, intensities were extracted using ImaGene software (BioDiscovery, El Segundo, CA). All extracted data were exported to Microsoft Excel (Microsoft Corporation, Redmond, WA) as tab delimited files. For each feature, a low intensity filter was introduced in which data were only included if the total back-ground-subtracted intensity of the two channels was greater than 400. We computed the log_2_ratio of Res-treated samples to untreated samples and assembled the ratio into a single tab-delimited file for each comparison. To perform statistical analysis, we imported each file to the Multiple Experiment Viewer (MeV) v4.0 .

To evaluate the significant deviation from zero, we compared the calculated log ratios using one-class Significance Analysis of Microarrays (SAM) . SAM was implemented with the maximum number of unique permutations available, and delta values were selected to give a median False Discovery Rate (10%). All other were set to the MeV default parameters. The obtained statistical significant parameters were subsequently filtered for a minimum fold change greater than 1.5. The genes are represented by multiple sets in both types of microarray platforms. We report the feature with the largest fold change for genes which were significantly differentially expressed.

**Pathway analysis**

We put the gene expression data into the Pathway Studio software [Rockville, MD] to identify molecular interactions. To determine the p-value associated with the biological processes, we firstly used Fisher's Exact Test as implemented by Pathway Studio. Subsequently, we applied Holm-Bonferroni method to adjust it for multiple hypothesis testing with an overall type-1 error rate of 0.05. To check the statistical significance of the key genes in the graphical output, we directly used custom-written Java code to implement Fisher's Exact Test, which shares the same parameters with the Holm-Bonferroni method. The Holm-Bonferroni adjusted p-values reported for the key genes evince the significance level for the number of connections to other genes in our data set, accounting for the number of known connections in the Pathway Studio database.

**Reference**

1. R. Jafari, H. Almqvist, H. Axelsson, M. Ignatushchenko, T. Lundbäck, P. Nordlund, D. M. Molina, The cellular thermal shift assay for evaluating drug target interactions in cells. *Nature protocols* **9**, 2100-2122 (2014).

2. A. Saeed, V. Sharov, J. White, J. Li, W. Liang, N. Bhagabati, J. Braisted, M. Klapa, T. Currier, M. Thiagarajan, TM4: a free, open-source system for microarray data management and analysis. *Biotechniques* **34**, 374-378 (2003).

3. V. G. Tusher, R. Tibshirani, G. Chu, Significance analysis of microarrays applied to the ionizing radiation response. *Proceedings of the National Academy of Sciences* **98**, 5116-5121 (2001).

4. S. Holm, A simple sequentially rejective multiple test procedure. *Scandinavian journal of statistics* **6**, 65-70 (1979).

**Supplementary Figures S1~S5**

**
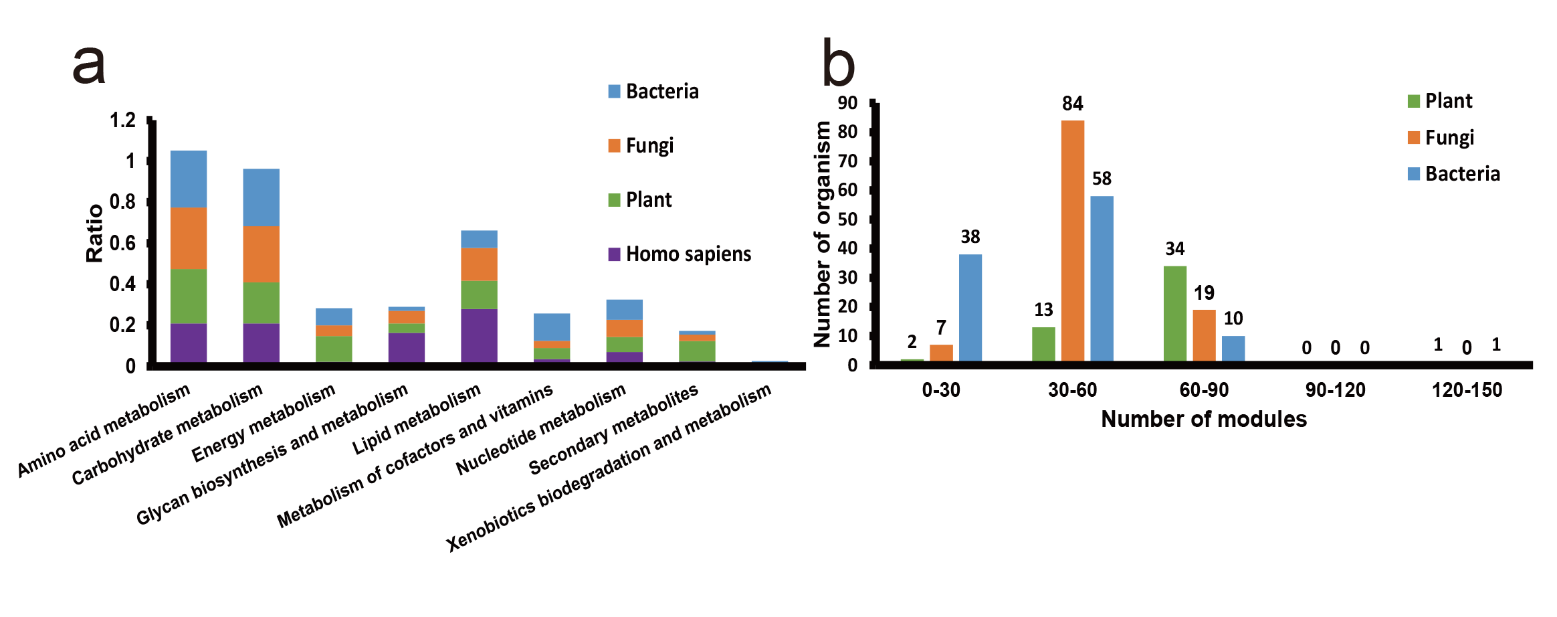
**

**Supplementary Figure S1. Function categories and number distribution of modules from human and plants/microbes.** (**a**) The proportion of different types of modules in human, plants, fungi and bacteria. The modules are classified into nine major groups based on the classification scheme proposed in KEGG pathway database. (**b**) The number of modules contained in organisms of plants, fungi and bacteria.

**
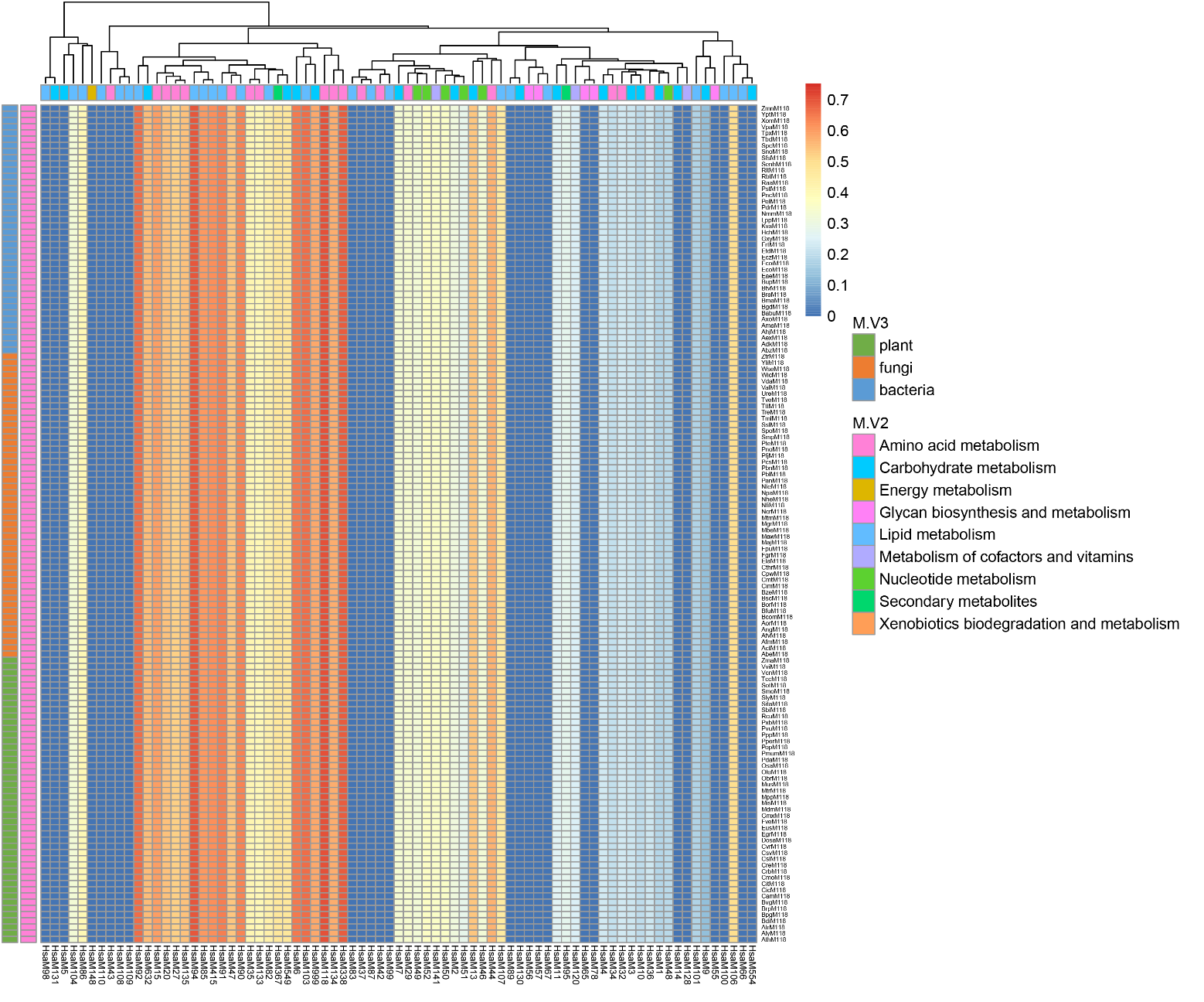
**

**Supplementary Figure S2.** Cluster of the module “M118: Glutathione biosynthesis” from 135 different organisms in plant/microbe.

**
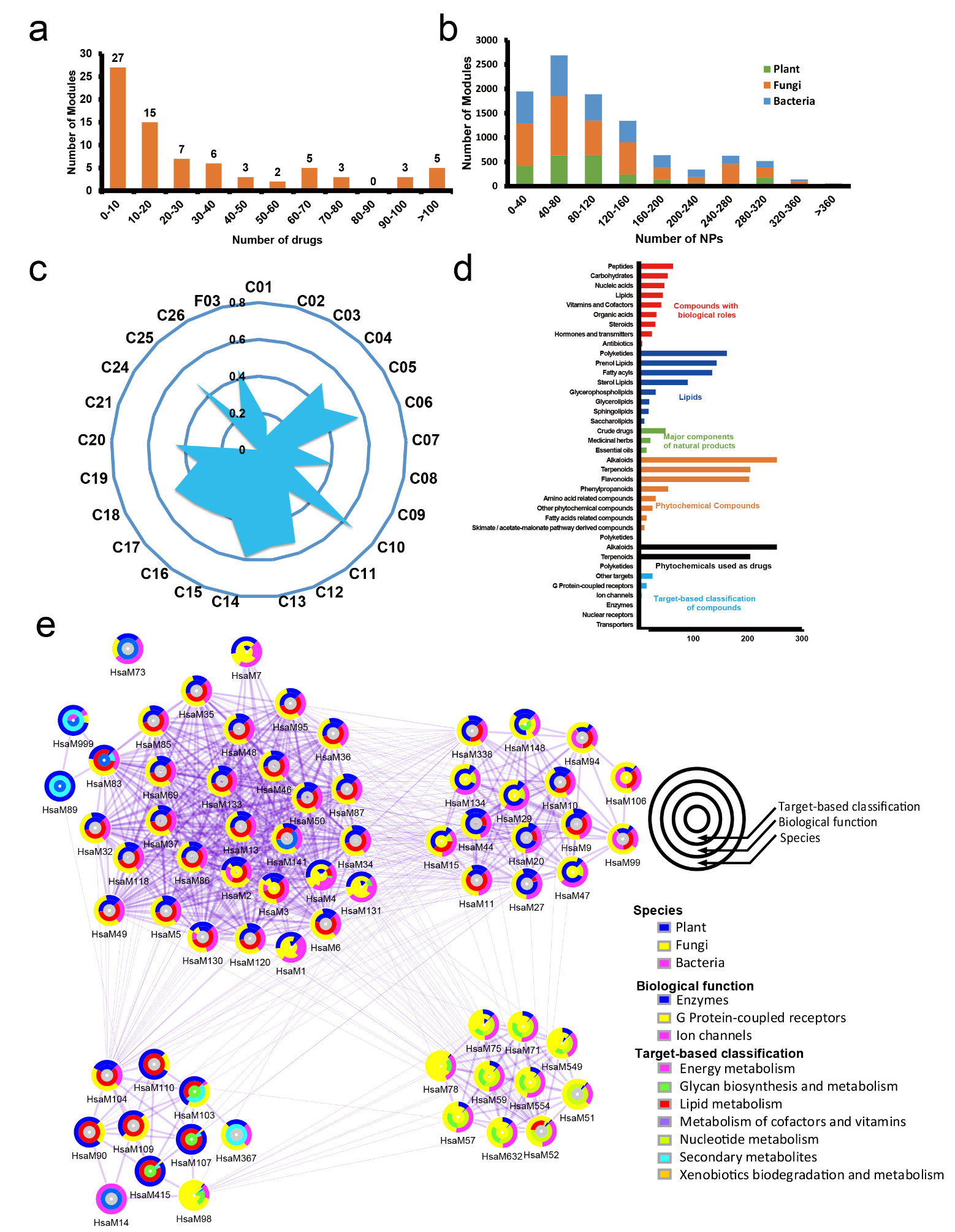
**

**Supplementary Figure S3. Drug and NP sets related to modules from human and plants/microbes.** (**a**) The number of drugs contained in human modules. (**b**) The number of NPs contained in plant/microbe modules. (**c**) A radar chart showing the disease categories treated by drugs related to the 87 human modules. Each spoke characterize one of the MeSH disease categories. The length of a spoke is the proportion of drugs which can treat diseases belong to a specific disease category. (**d**) The number of NPs related to plant/microbe modules in the 6 different compound categories based on the KEGG COMPOUND classification system. (**e**) The network connecting human modules based on their shared plant/microbe modules whose related NPs have high structure similarity (*P*-value<0.01) with drugs targeting those human modules.

**
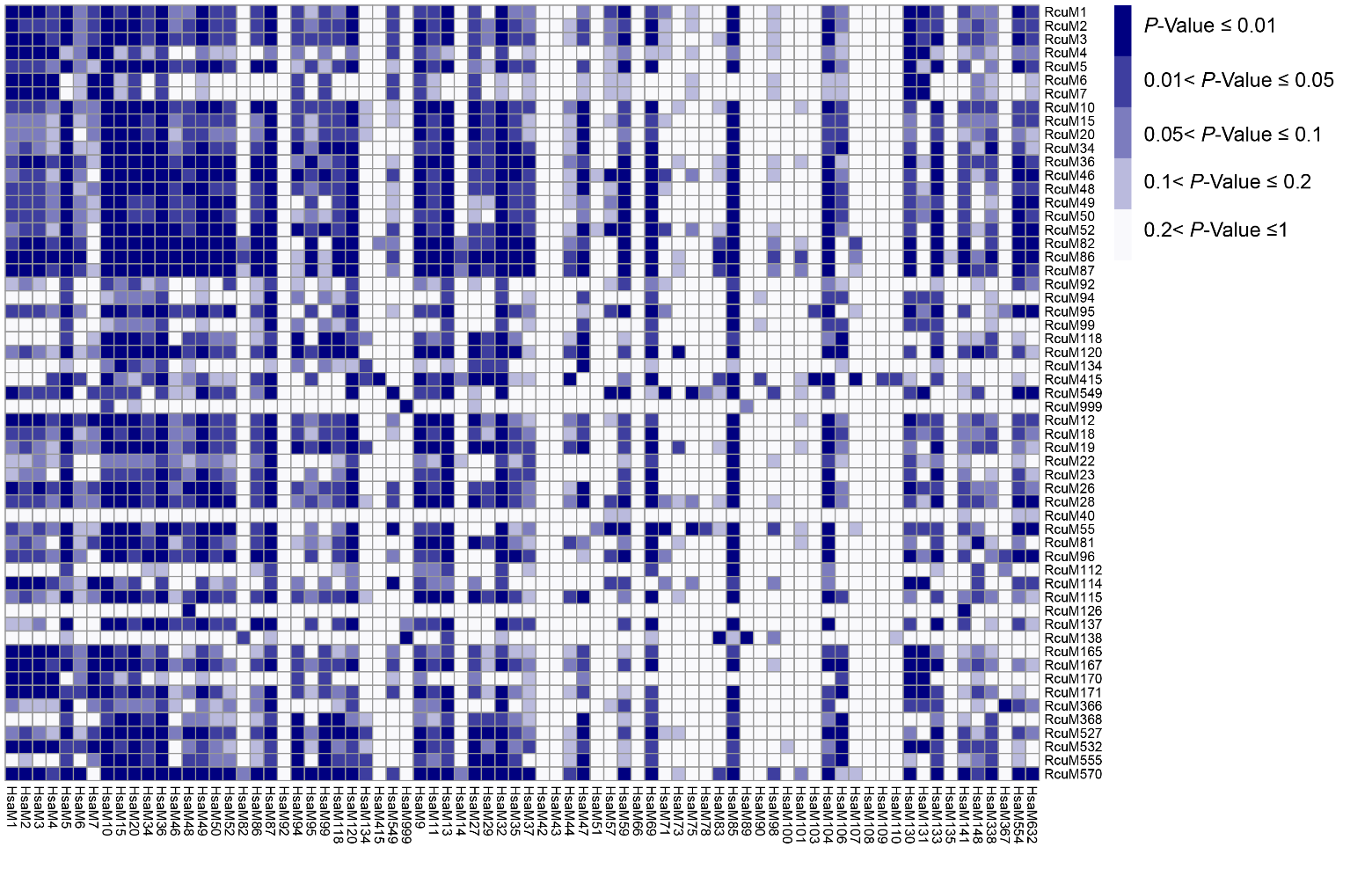
**

**Supplementary Figure S4.** The chemical structure similarity matrix between drug and NP sets of human and *Ricinus communis* modules.

**
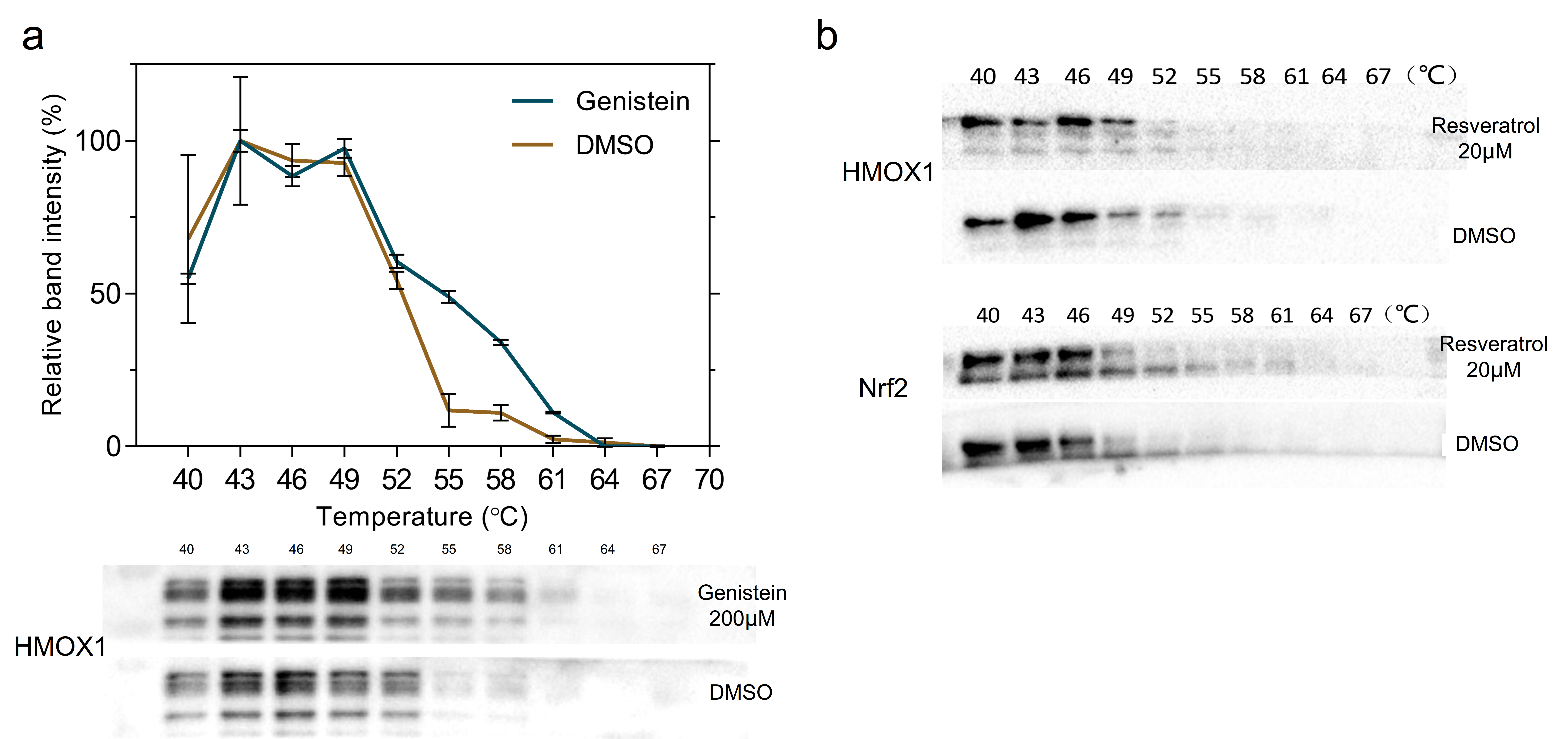
**

**Supplementary Figure S5.** The CETSA experiment shows that (**a**) genistein can directly bind to the target protein HMOX1, (**b**) while resveratrol cannot bind to HMOX1 and Nrf2.
