# Supplementary material for: New strategy for drug discovery by large-scale association analysis of molecular networks of different species
Source: Sci Rep. 2016 Feb 25;6:21872. doi: 10.1038/srep21872 (PMC4766474; doi:10.1038/srep21872)
Supplement: Supplementary Table S2 [file srep21872-s3.doc]

**Supplementary Table S2. Top 25 drug-like plant/microbe modules**

| **Species** | **Module ID** | **Module Name** | **NPs In KEGG (Drugs In KEGG, Drugbank or TTD)** |
| --- | --- | --- | --- |
| Abp | AbpM86 | beta-Oxidation, acyl-CoA synthesis | C00010**(DB01992)**; C00022**(DB00119)**; C00024; C00025**(D00007)**; C00026; C00042**(DAP000545)**; C00091**(DB03699)**; C00122**(D02308)**; C00136; C00154; C00164**(DB01762)**; C00232; C00249**(D05341)**; C00332**(DB03059)**; C00334**(D00058)**; C00356; C00489**(DB03553)**; C00517**(DB03381)**; C00527; C00877; C01144; C01832; C01944; C02411; C02593**(DB02180)**; C03221; C05258; C05259; C05260; C05261; C05262; C05263; C05264(DB03192); C05265; C05266; C05267; C05268; C05269; C05270; C05271; C05272; C05273; C05274; C05275; C05276; C05279; C05280; C06010; |
| Abv | AbvM86 | beta-Oxidation, acyl-CoA synthesis | C00010**(DB01992)**; C00022**(DB00119)**; C00024; C00025**(D00007)**; C00026; C00042**(DAP000545)**; C00091**(DB03699)**; C00122**(D02308)**; C00136; C00154; C00164**(DB01762)**; C00232; C00249**(D05341)**; C00332**(DB03059)**; C00334**(D00058)**; C00356; C00489**(DB03553)**; C00517**(DB03381)**; C00527; C00877; C01144; C01832; C01944; C02411; C02593**(DB02180)**; C03221; C05258; C05259; C05260; C05261; C05262; C05263; C05264(DB03192); C05265; C05266; C05267; C05268; C05269; C05270; C05271; C05272; C05273; C05274; C05275; C05276; C05279; C05280; C06010; |
| Aje | AjeM86 | beta-Oxidation, acyl-CoA synthesis | C00010**(DB01992)**; C00022**(DB00119)**; C00024; C00025**(D00007)**; C00026; C00042**(DAP000545)**; C00091**(DB03699)**; C00122**(D02308)**; C00136; C00154; C00164**(DB01762)**; C00232; C00249**(D05341)**; C00332**(DB03059)**; C00334**(D00058)**; C00356; C00489**(DB03553)**; C00517**(DB03381)**; C00527; C00877; C01144; C01832; C01944; C02411; C02593**(DB02180)**; C03221; C05258; C05259; C05260; C05261; C05262; C05263; C05264(DB03192); C05265; C05266; C05267; C05268; C05269; C05270; C05271; C05272; C05273; C05274; C05275; C05276; C05279; C05280; C06010; |
| Babu | BabuM86 | beta-Oxidation, acyl-CoA synthesis | C00010**(DB01992)**; C00022**(DB00119)**; C00024; C00025**(D00007)**; C00026; C00042**(DAP000545)**; C00091**(DB03699)**; C00122**(D02308)**; C00136; C00154; C00164**(DB01762)**; C00232; C00249**(D05341)**; C00332**(DB03059)**; C00334**(D00058)**; C00356; C00489**(DB03553)**; C00527; C00877; C01089; C01144; C02411; C03221; C03561; C05258; C05259; C05260; C05261; C05262; C05263; C05264(DB03192); C05266; C05267; C05268; C05269; C05271; C05272; C05273; C05275; C05276; C06010; |
| Cci | CciM86 | beta-Oxidation, acyl-CoA synthesis | C00010**(DB01992)**; C00022**(DB00119)**; C00024; C00025**(D00007)**; C00026; C00042**(DAP000545)**; C00091**(DB03699)**; C00122**(D02308)**; C00136; C00154; C00164**(DB01762)**; C00232; C00249**(D05341)**; C00332**(DB03059)**; C00334**(D00058)**; C00356; C00489**(DB03553)**; C00517**(DB03381)**; C00527; C00877; C01144; C01832; C01944; C02411; C02593**(DB02180)**; C03221; C05258; C05259; C05260; C05261; C05262; C05263; C05264(DB03192); C05265; C05266; C05267; C05268; C05269; C05270; C05271; C05272; C05273; C05274; C05275; C05276; C05279; C05280; C06010; |
| Cput | CputM86 | beta-Oxidation, acyl-CoA synthesis | C00010**(DB01992)**; C00022**(DB00119)**; C00024; C00025**(D00007)**; C00026; C00042**(DAP000545)**; C00091**(DB03699)**; C00122**(D02308)**; C00136; C00154; C00164**(DB01762)**; C00232; C00249**(D05341)**; C00332**(DB03059)**; C00334**(D00058)**; C00356; C00489**(DB03553)**; C00517**(DB03381)**; C00527; C00877; C01144; C01832; C01944; C02411; C02593**(DB02180)**; C03221; C05258; C05259; C05260; C05261; C05262; C05263; C05264(DB03192); C05265; C05266; C05267; C05268; C05269; C05270; C05271; C05272; C05273; C05274; C05275; C05276; C05279; C05280; C06010; |
| Cthr | CthrM86 | beta-Oxidation, acyl-CoA synthesis | C00010**(DB01992)**; C00022**(DB00119)**; C00024; C00025**(D00007)**; C00026; C00042**(DAP000545)**; C00091**(DB03699)**; C00122**(D02308)**; C00136; C00154; C00164**(DB01762)**; C00232; C00249**(D05341)**; C00332**(DB03059)**; C00334**(D00058)**; C00356; C00489**(DB03553)**; C00517**(DB03381)**; C00527; C00877; C01144; C01832; C01944; C02411; C02593**(DB02180)**; C03221; C05258; C05259; C05260; C05261; C05262; C05263; C05264(DB03192); C05265; C05266; C05267; C05268; C05269; C05270; C05271; C05272; C05273; C05274; C05275; C05276; C05279; C05280; C06010; |
| Dti | DtiM86 | beta-Oxidation, acyl-CoA synthesis | C00010**(DB01992)**; C00022**(DB00119)**; C00024; C00025**(D00007)**; C00026; C00042**(DAP000545)**; C00091**(DB03699)**; C00122**(D02308)**; C00136; C00154; C00164**(DB01762)**; C00232; C00249**(D05341)**; C00332**(DB03059)**; C00334**(D00058)**; C00356; C00489**(DB03553)**; C00527; C00741; C00810; C00877; C00989**(DB01440)**; C01089; C01144; C01769**(DB04364)**; C02331; C02411; C02630; C03044**(DB02418)**; C03058; C03221; C03561; C05258; C05259; C05260; C05261; C05262; C05263; C05264(DB03192); C05265; C05266; C05267; C05268; C05269; C05271; C05272; C05273; C05275; C05276; C06010; C11062; C20657; |
| Eol | EolM86 | beta-Oxidation, acyl-CoA synthesis | C00010**(DB01992)**; C00022**(DB00119)**; C00024; C00025**(D00007)**; C00026; C00042**(DAP000545)**; C00091**(DB03699)**; C00122**(D02308)**; C00136; C00154; C00164**(DB01762)**; C00232; C00249**(D05341)**; C00332**(DB03059)**; C00334**(D00058)**; C00356; C00489**(DB03553)**; C00527; C00877; C01144; C01832; C01944; C02411; C02593**(DB02180)**; C03221; C03547**(DB02418)**; C03561; C05258; C05259; C05260; C05261; C05262; C05263; C05264(DB03192); C05265; C05266; C05267; C05268; C05269; C05270; C05271; C05272; C05273; C05274; C05275; C05276; C06010; |
| Gba | GbaM86 | beta-Oxidation, acyl-CoA synthesis | C00010**(DB01992)**; C00022**(DB00119)**; C00024; C00025**(D00007)**; C00026; C00042**(DAP000545)**; C00091**(DB03699)**; C00122**(D02308)**; C00136; C00154; C00164**(DB01762)**; C00232; C00249**(D05341)**; C00332**(DB03059)**; C00334**(D00058)**; C00356; C00489**(DB03553)**; C00527; C00877; C01144; C01384**(DB04299)**; C02411; C03221; C03561; C05258; C05259; C05260; C05261; C05262; C05263; C05264(DB03192); C05265; C05266; C05267; C05268; C05269; C05271; C05272; C05273; C05275; C05276; C06010; |
| Gsl | GslM86 | beta-Oxidation, acyl-CoA synthesis | C00010**(DB01992)**; C00022**(DB00119)**; C00024; C00025**(D00007)**; C00026; C00042**(DAP000545)**; C00091**(DB03699)**; C00122**(D02308)**; C00136; C00154; C00164**(DB01762)**; C00232; C00249**(D05341)**; C00332**(DB03059)**; C00334**(D00058)**; C00356; C00527; C00877; C01144; C01832; C01944; C02411; C02593**(DB02180)**; C02630; C03221; C05258; C05259; C05260; C05261; C05262; C05263; C05264(DB03192); C05265; C05266; C05267; C05268; C05269; C05270; C05271; C05272; C05273; C05274; C05275; C05276; C06010; |
| Hch | HchM86 | beta-Oxidation, acyl-CoA synthesis | C00010**(DB01992)**; C00022**(DB00119)**; C00024; C00025**(D00007)**; C00026; C00042**(DAP000545)**; C00091**(DB03699)**; C00122**(D02308)**; C00136; C00154; C00164**(DB01762)**; C00249**(D05341)**; C00332**(DB03059)**; C00334**(D00058)**; C00356; C00527; C00877; C01144; C01412; C01832; C01944; C02411; C02593**(DB02180)**; C03221; C03547**(DB02418)**; C03561; C05258; C05259; C05260; C05261; C05262; C05263; C05264(DB03192); C05265; C05266; C05267; C05268; C05269; C05270; C05271; C05272; C05273; C05274; C05275; C05276; C05279; C05280; C06010; C06142; |
| Lbc | LbcM86 | beta-Oxidation, acyl-CoA synthesis | C00010**(DB01992)**; C00022**(DB00119)**; C00024; C00025**(D00007)**; C00026; C00042**(DAP000545)**; C00091**(DB03699)**; C00122**(D02308)**; C00136; C00154; C00164**(DB01762)**; C00232; C00249**(D05341)**; C00332**(DB03059)**; C00334**(D00058)**; C00356; C00489**(DB03553)**; C00517**(DB03381)**; C00527; C00877; C01144; C01832; C01944; C02411; C02593**(DB02180)**; C03221; C05258; C05259; C05260; C05261; C05262; C05263; C05264(DB03192); C05265; C05266; C05267; C05268; C05269; C05270; C05271; C05272; C05273; C05274; C05275; C05276; C05279; C05280; C06010; |
| Pbl | PblM86 | beta-Oxidation, acyl-CoA synthesis | C00010**(DB01992)**; C00022**(DB00119)**; C00024; C00025**(D00007)**; C00026; C00042**(DAP000545)**; C00091**(DB03699)**; C00122**(D02308)**; C00136; C00154; C00164**(DB01762)**; C00232; C00249**(D05341)**; C00332**(DB03059)**; C00334**(D00058)**; C00356; C00489**(DB03553)**; C00517**(DB03381)**; C00527; C00877; C01144; C01832; C01944; C02411; C02593**(DB02180)**; C03221; C05258; C05259; C05260; C05261; C05262; C05263; C05264(DB03192); C05265; C05266; C05267; C05268; C05269; C05270; C05271; C05272; C05273; C05274; C05275; C05276; C05279; C05280; C06010; |
| Pbn | PbnM86 | beta-Oxidation, acyl-CoA synthesis | C00010**(DB01992)**; C00022**(DB00119)**; C00024; C00025**(D00007)**; C00026; C00042**(DAP000545)**; C00091**(DB03699)**; C00122**(D02308)**; C00136; C00154; C00164**(DB01762)**; C00232; C00249**(D05341)**; C00332**(DB03059)**; C00334**(D00058)**; C00356; C00489**(DB03553)**; C00517**(DB03381)**; C00527; C00877; C01144; C01832; C01944; C02411; C02593**(DB02180)**; C03221; C05258; C05259; C05260; C05261; C05262; C05263; C05264(DB03192); C05265; C05266; C05267; C05268; C05269; C05270; C05271; C05272; C05273; C05274; C05275; C05276; C05279; C05280; C06010; |
| Pdr | PdrM86 | beta-Oxidation, acyl-CoA synthesis | C00010**(DB01992)**; C00022**(DB00119)**; C00024; C00025**(D00007)**; C00026; C00042**(DAP000545)**; C00091**(DB03699)**; C00122**(D02308)**; C00136; C00154; C00164**(DB01762)**; C00232; C00249**(D05341)**; C00332**(DB03059)**; C00334**(D00058)**; C00356; C00489**(DB03553)**; C00527; C00877; C01089; C01144; C01832; C01944; C02411; C02593**(DB02180)**; C02630; C03058; C03221; C03547**(DB02418)**; C03561; C05258; C05259; C05260; C05261; C05262; C05263; C05264(DB03192); C05265; C05266; C05267; C05268; C05269; C05270; C05271; C05272; C05273; C05274; C05275; C05276; C05279; C05280; C06010; |
| Pst | PstM86 | beta-Oxidation, acyl-CoA synthesis | C00010**(DB01992)**; C00022**(DB00119)**; C00024; C00025**(D00007)**; C00026; C00042**(DAP000545)**; C00091**(DB03699)**; C00122**(D02308)**; C00136; C00154; C00164**(DB01762)**; C00232; C00249**(D05341)**; C00332**(DB03059)**; C00334**(D00058)**; C00356; C00489**(DB03553)**; C00497; C00527; C00877; C01144; C01412; C01832; C01944; C02411; C02593**(DB02180)**; C02630; C03058; C03221; C03561; C05258; C05259; C05260; C05261; C05262; C05263; C05264(DB03192); C05265; C05266; C05267; C05268; C05269; C05270; C05271; C05272; C05273; C05274; C05275; C05276; C05279; C05280; C06010; C06142; |
| Rae | RaeM86 | beta-Oxidation, acyl-CoA synthesis | C00010**(DB01992)**; C00022**(DB00119)**; C00024; C00025**(D00007)**; C00026; C00042**(DAP000545)**; C00091**(DB03699)**; C00122**(D02308)**; C00136; C00154; C00164**(DB01762)**; C00232; C00249**(D05341)**; C00332**(DB03059)**; C00334**(D00058)**; C00356; C00489**(DB03553)**; C00527; C00877; C01144; C01832; C01944; C02411; C02593**(DB02180)**; C05258; C05259; C05260; C05261; C05262; C05263; C05264(DB03192); C05265; C05266; C05267; C05268; C05269; C05270; C05274; |
| Sct | SctM86 | beta-Oxidation, acyl-CoA synthesis | C00010**(DB01992)**; C00022**(DB00119)**; C00024; C00025**(D00007)**; C00026; C00042**(DAP000545)**; C00091**(DB03699)**; C00122**(D02308)**; C00136; C00154; C00164**(DB01762)**; C00232; C00249**(D05341)**; C00332**(DB03059)**; C00334**(D00058)**; C00356; C00489**(DB03553)**; C00527; C00877; C01089; C01144; C01384**(DB04299)**; C01832; C01944; C02411; C02593**(DB02180)**; C03221; C03561; C05102; C05258; C05259; C05260; C05261; C05262; C05263; C05264(DB03192); C05266; C05267; C05268; C05269; C05270; C05271; C05272; C05273; C05274; C05275; C05276; C06010; |
| Sla | SlaM86 | beta-Oxidation, acyl-CoA synthesis | C00010**(DB01992)**; C00022**(DB00119)**; C00024; C00025**(D00007)**; C00026; C00042**(DAP000545)**; C00091**(DB03699)**; C00122**(D02308)**; C00136; C00154; C00164**(DB01762)**; C00232; C00249**(D05341)**; C00332**(DB03059)**; C00334**(D00058)**; C00356; C00489**(DB03553)**; C00517**(DB03381)**; C00527; C00877; C01144; C01832; C01944; C02411; C02593**(DB02180)**; C03221; C05258; C05259; C05260; C05261; C05262; C05263; C05264(DB03192); C05265; C05266; C05267; C05268; C05269; C05270; C05271; C05272; C05273; C05274; C05275; C05276; C05279; C05280; C06010; |
| Spc | SpcM86 | beta-Oxidation, acyl-CoA synthesis | C00010**(DB01992)**; C00022**(DB00119)**; C00024; C00025**(D00007)**; C00026; C00042**(DAP000545)**; C00091**(DB03699)**; C00122**(D02308)**; C00136; C00154; C00164**(DB01762)**; C00232; C00249**(D05341)**; C00332**(DB03059)**; C00334**(D00058)**; C00356; C00489**(DB03553)**; C00527; C00877; C01144; C01412; C01832; C01944; C02593**(DB02180)**; C03221; C03561; C05258; C05259; C05260; C05261; C05262; C05263; C05264(DB03192); C05265; C05266; C05267; C05268; C05269; C05270; C05271; C05272; C05273; C05274; C05275; C05276; C05279; C05280; C06010; |
| Tbi | TbiM86 | beta-Oxidation, acyl-CoA synthesis | C00010**(DB01992)**; C00022**(DB00119)**; C00024; C00025**(D00007)**; C00026; C00042**(DAP000545)**; C00091**(DB03699)**; C00122**(D02308)**; C00136; C00154; C00164**(DB01762)**; C00232; C00249**(D05341)**; C00332**(DB03059)**; C00334**(D00058)**; C00356; C00489**(DB03553)**; C00527; C00877; C01144; C01832; C01944; C02411; C02593**(DB02180)**; C02630; C03058; C03221; C05258; C05259; C05260; C05261; C05262; C05263; C05264(DB03192); C05265; C05266; C05267; C05268; C05269; C05270; C05271; C05272; C05273; C05274; C05275; C05276; C06010; |
| Tml | TmlM86 | beta-Oxidation, acyl-CoA synthesis | C00010**(DB01992)**; C00022**(DB00119)**; C00024; C00025**(D00007)**; C00026; C00042**(DAP000545)**; C00091**(DB03699)**; C00122**(D02308)**; C00136; C00154; C00164**(DB01762)**; C00232; C00249**(D05341)**; C00332**(DB03059)**; C00334**(D00058)**; C00356; C00489**(DB03553)**; C00517**(DB03381)**; C00527; C00877; C01144; C01832; C01944; C02411; C02593**(DB02180)**; C03221; C05258; C05259; C05260; C05261; C05262; C05263; C05264(DB03192); C05265; C05266; C05267; C05268; C05269; C05270; C05271; C05272; C05273; C05274; C05275; C05276; C05279; C05280; C06010; |
| Tra | TraM86 | beta-Oxidation, acyl-CoA synthesis | C00010**(DB01992)**; C00022**(DB00119)**; C00024; C00025**(D00007)**; C00026; C00042**(DAP000545)**; C00091**(DB03699)**; C00122**(D02308)**; C00136; C00154; C00164**(DB01762)**; C00232; C00249**(D05341)**; C00332**(DB03059)**; C00334**(D00058)**; C00356; C00489**(DB03553)**; C00527; C00877; C01089; C01144; C01832; C01944; C02411; C02593**(DB02180)**; C03221; C03561; C05258; C05259; C05260; C05261; C05262; C05263; C05264(DB03192); C05265; C05266; C05267; C05268; C05269; C05270; C05271; C05272; C05273; C05274; C05275; C05276; C06010; |
| Yli | YliM86 | beta-Oxidation, acyl-CoA synthesis | C00010**(DB01992)**; C00022**(DB00119)**; C00024; C00025**(D00007)**; C00026; C00042**(DAP000545)**; C00091**(DB03699)**; C00122**(D02308)**; C00136; C00154; C00164**(DB01762)**; C00232; C00249**(D05341)**; C00332**(DB03059)**; C00334**(D00058)**; C00356; C00489**(DB03553)**; C00517**(DB03381)**; C00527; C00877; C01144; C01832; C01944; C02411; C02593**(DB02180)**; C03221; C05258; C05259; C05260; C05261; C05262; C05263; C05264(DB03192); C05265; C05266; C05267; C05268; C05269; C05270; C05271; C05272; C05273; C05274; C05275; C05276; C05280; C06010; |
